# Cryptic Diversity of Black Band Disease Cyanobacteria in *Siderastrea siderea* Corals Revealed by Chemical Ecology and Comparative Genome-Resolved Metagenomics

**DOI:** 10.3390/md21020076

**Published:** 2023-01-22

**Authors:** Julie L. Meyer, Sarath P. Gunasekera, Anya L. Brown, Yousong Ding, Stephanie Miller, Max Teplitski, Valerie J. Paul

**Affiliations:** 1 Department of Soil, Water, and Ecosystem Sciences, University of Florida, Gainesville, FL 32610, USA; 2 Smithsonian Marine Station, Ft. Pierce, FL 34949, USA; 3 School of Natural Resources and Environment, University of Florida, Gainesville, FL 32603, USA; 4 Department of Evolution and Ecology & Bodega Marine Lab, University of California, Bodega Bay, CA 94923, USA; 5 Department of Medicinal Chemistry & Center for Natural Products, Drug Discovery and Development, University of Florida, Gainesville, FL 32603, USA

**Keywords:** *Roseofilum*, looekeyolide, malyngamide, lasso peptide, metagenome-assembled genomes, biosynthetic gene cluster

## Abstract

Black band disease is a globally distributed and easily recognizable coral disease. Despite years of study, the etiology of this coral disease, which impacts dozens of stony coral species, is not completely understood. Although black band disease mats are predominantly composed of the cyanobacterial species *Roseofilum reptotaenium*, other filamentous cyanobacterial strains and bacterial heterotrophs are readily detected. Through chemical ecology and metagenomic sequencing, we uncovered cryptic strains of *Roseofilum* species from *Siderastrea siderea* corals that differ from those on other corals in the Caribbean and Pacific. Isolation of metabolites from *Siderastrea*-derived *Roseofilum* revealed the prevalence of unique forms of looekeyolides, distinct from previously characterized *Roseofilum reptotaenium* strains. In addition, comparative genomics of *Roseofilum* strains showed that only *Siderastrea*-based *Roseofilum* strains have the genetic capacity to produce lasso peptides, a family of compounds with diverse biological activity. All nine *Roseofilum* strains examined here shared the genetic capacity to produce looekeyolides and malyngamides, suggesting these compounds support the ecology of this genus. Similar biosynthetic gene clusters are not found in other cyanobacterial genera associated with black band disease, which may suggest that looekeyolides and malyngamides contribute to disease etiology through yet unknown mechanisms.

## 1. Introduction

Breakthroughs in sequencing technologies over the last few decades have shed light on the extensive genetic diversity of microbial life and its tremendous wealth of biosynthetic gene clusters. Cyanobacteria, especially filamentous types, have proven to be a rich source of secondary metabolites, including antimicrobial and bioactive natural products [1,2,3]. In some cases, these unique products are toxic and can be produced at levels high enough to be detrimental to humans, pets, and wildlife such as during harmful algal blooms of the cyanobacteria *Microcystis*. Recent metagenomic and metatranscriptomic sequencing of *Microcystis* blooms revealed the presence not only of co-occurring toxigenic and non-toxigenic strains, but also strains that harbored partial gene clusters for microcystin that were abundant and expressed during specific successional phases of the bloom [4]. Thus, the genomic revolution is providing new avenues to explore the functional and ecological roles of cryptic diversity within cyanobacteria.

Black band disease (BBD) is arguably the longest-studied coral disease, as it was first identified in the scientific literature in the 1970s [5] and documented in artwork as early as the 1890s [6]. Yet, we still do not fully understand the etiology of this destructive and globally distributed coral disease. The engineer of BBD is the filamentous cyanobacterium *Roseofilum reptotaenium* [7], which forms a dense, polymicrobial mat under which anoxic and sulfidic conditions smother and kill coral tissue [8]. While *Roseofilum* is part of the normal microflora of corals, where it can be found at low levels even in corals unaffected by BBD [9], it is unknown what triggers *Roseofilum* to form BBD mats. However, the natural products formed by *Roseofilum* may play a role in manipulating the microbial communities on the coral surface through quorum sensing [9] or other means.

While investigating natural products from BBD mats in corals, we uncovered a pair of novel compounds related to previously described looekeyolides A and B [10]. Strikingly, these compounds were detected only in BBD cyanobacterial mats collected from the massive starlet coral, *Siderastrea siderea*. Herein, we characterized the cyanobacteria associated with BBD in *S. siderea* corals through chemical ecology, 16S rRNA sequencing, and genome-resolved metagenomics to determine the differences between these cyanobacteria and previously characterized strains of *Roseofilum*.

## 2. Results

### 2.1. Collection of S. siderea-Associated Black Band Disease Cyanobacterial Mats

BBD cyanobacterial mats were sampled from *S. siderea* corals when found during SCUBA diving expeditions in Belize and Florida from 2014 to 2018 (Table S2). These samples were divided among analyses for characterization of major secondary metabolites, characterization of bacterial community composition, and genome-resolved metagenomics. In addition, a non-axenic, cyanobacterial enrichment culture was grown from a BBD mat on a *S. siderea* coral in Florida. The predominant cyanobacterium was likely a *Geitlerinema* strain, as this was the only cyanobacterial genome retrieved from the culture (Table S2).

### 2.2. Isolation and Characterization of Novel Looekeyolides

**Figure 1:**
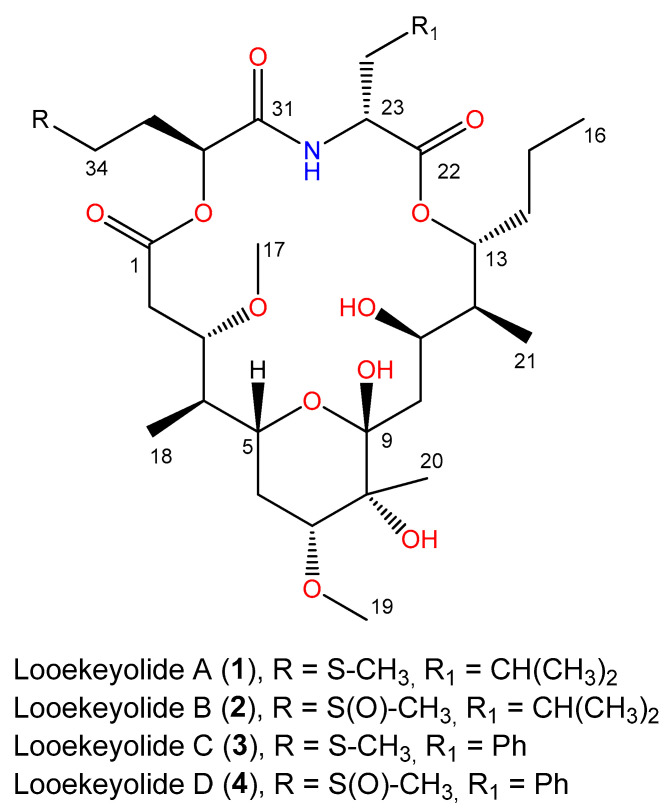
Chemical structures of previously isolated looekeyolides A and B and the new looekeyolides C and D described in this study.

Previously, we reported two related macrocyclic metabolites, looekeyolide A and looekeyolide B (Figure 1), isolated from the lipophilic extracts of black band disease mats collected from *Montastraea cavernosa*, *Orbicella annularis*, *Orbicella faveolata*, *Pseudodiploria strigosa*, and *Goniopora fruticosa*, and from cultured *Roseofilum reptotaenium* [10]. Looekeyolide A is a 20-membered macrocyclic compound formed by a 16-carbon polyketide chain, 2-deamino-2-hydroxymethionine and d-leucine, and looekeyolide B is its auto-oxidation product at the 2-deamino-2-hydroxymethionine moiety. Interestingly, liquid chromatography-mass spectrometry (LC-MS) analysis of the BBD extracts from a collection of several small samples from the coral *S. siderea* growing in Southwater Caye, Belize, collected in July 2014 indicated the absence of the peaks at *m*/*z* 686 [M + Na]^+^ for looekeyolide A and at *m*/*z* 702 [M + Na]^+^ for looekeyolide B. Instead, we observed peaks at *m*/*z* 720 [M + Na]^+^ for looekeyolide C and 736 [M + Na]^+^ for looekeyolide D, two new related compounds separated by 16 mass units (Figure 1, Figure S1) as reported for looekeyolides A and B. Similar results were observed from the lipophilic extract of an August 2018 BBD collection from *S. siderea* growing in Curlew Cay, Belize (Figure S2) and the lipophilic extract of a July 2018 BBD collection from *S. siderea* from Fort Lauderdale, Florida (Figure S3). This mass spectral information prompted us to conduct further chemical investigation of these small samples.

The lipophilic extracts of each batch of freeze-dried sample collections were subjected to reversed phase column chromatography followed by reversed phase high performance liquid chromatography (HPLC) using MeOH-20% water to give looekeyolide D. Although the related compound looekeyolide C was detected in low-resolution electrospray ionization mass spectrometry (LRESIMS) traces, the isolation procedures auto-oxidized it completely to the stable looekeyolide D, and looekeyolide C was not isolated for other spectral studies.

Looekeyolide D (**4**) was obtained as a white powder; [α]^25^D 6.0° (*c* 0.22, MeOH); UV (MeOH) λ_max_ (log *ε*) 225 (3.77); ^1^H NMR, ^13^C NMR, double quantum filtered correlated spectroscopy (DQF COSY), heteronuclear multiple bond correlation (HMBC), and nuclear Overhauser effect spectroscopy (NOESY) data, see Table 1; high resolution electrospray ionization mass spectrometry (HRESI)/atmospheric-pressure chemical ionization mass spectrometry (APCIMS) *m*/*z* 736.3362 [M + Na]^+^ (calcd for C_35_H_55_NO_12_SNa, 702.33337) supported the molecular formula of C_35_H_55_NO_12_S. The ^1^H and ^13^C NMR spectral data were indicative of one α-hydroxy acid, one α-amino acid, and one highly substituted 16-carbon polyketide chain in the molecule (Table 1). Complete NMR analysis of ^1^H, ^13^C, DQF COSY, edited heteronuclear single quantum coherence spectroscopy (HSQC) and HMBC data for C-22 to C-30 identified this fragment as phenylalanine. The fragment C-31 to C-34 indicated doubling of ^1^H signals as overlapped multiplets for H_2_-34 (*δ*_H_ 2.58, 2.46) and the split methyl signal H_3_-35 (*δ*_H_ 2.508, 2.501). The expected doubling of the ^13^C signals for C-31 to C-34 as in looekeyolide B (**2**) were not clearly visible due to weak spectrum. This information, together with the presence of a sulfur atom in the molecular formula, suggested the presence of a hydroxymethionine sulfoxide residue in the molecule as in the related lookeyolide B. The COSY spectrum indicated coupling of hydroxymethine H-32 (*δ*_H_ 4.86, *δ*_C_ 73.6) to methylenes H_2_-33 (*δ*_H_ 1.83, *δ*_C_ 25.4) and then, in turn, to H_2_-34 (*δ*_H_ 2.58, 2.46, *δ*_C_ 48.9). The HMBC spectrum showed correlation of methylene H_2_-34 to methyl carbon signal C-35 (*δ*_C_ 37.3) and the methyl split-singlet-S-35-H_3_ (*δ*_H_ 2.508/2.501), in turn, to C-34, (*δ*_C_ 48.9). These data confirmed the presence of a hydroxymethionine sulfoxide residue in the molecule. Following the interpretation of DQF COSY, edited HSQC and ^13^C experiments, the remaining ^1^H signals were assignable to two partial structures C-2 to C-7, C-10 to C-16, two *O-*Me groups (H-17, *δ*_H_ 3.32; H-19, *δ*_H_ 3.38), five hydroxyl methines (H-3, *δ*_H_ 4.34; H-5, *δ*_H_ 3.60; H-7, *δ*_H_ 3.44; H-11, *δ*_H_ 4.70; H-13, *δ*_H_ 4.99), four methyl groups (C-16, *δ*_H_ 0.93, *δ*_C_ 14.3; C-18, *δ*_H_ 0.81, *δ*_C_ 9.7; C-20, *δ*_H_ 1.25, *δ*_C_ 19.6; C-21, *δ*_H_ 0.98, *δ*_C_ 11.9), two quaternary carbons (C-8, *δ*_C_ 74.7; C-9, *δ*_C_ 102.0) and one ester carbonyl group (C-1, *δ*_C_ 173.4) (Table 1). HMBC correlations from H-3 (*δ*_H_ 4.34) and H_2_-2 (*δ*_H_ 2.52 and 2.36) to C-1 (*δ*_C_ 173.4) connected the remaining ester carbonyl. The weak HMBC data did not show correlation from H_3_-17 (*δ*_H_ 3.32) to C-3 (*δ*_C_ 77.6) and H_3_-19 (*δ*_H_ 3.38) to C-7 (*δ*_C_ 79.5) but the ^1^H and ^13^C chemical shift values, similar to those reported for **2** and the NOE data observed between H-4 and H-17 and H-20 and H-19, connected the two *O*Me groups to the C-3 and C-7 positions. HMBC correlations from H_3_-18 methyl (*δ*_H_ 0.81) to C-3 (*δ*_C_ 77.6) and to C-5 (*δ*_C_ 70.8) connected this methyl group to C-4 position. These data connected the methyl group, two *O*Me groups and the carbonyl group to the C-2 to C-7 partial structure. HMBC correlations indicated H_3_-20 (*δ*_H_ 1.25) to C-7 (*δ*_C_ 80.0), C-8 (*δ*_C_ 74.7), and C-9 (*δ*_C_ 102.0), H_2_-6 (*δ*_H_ 2.08, 1.42) to C-5 (*δ*_C_ 70.8) and C-7 (*δ*_C_ 80.0), H-10a (*δ*_H_ 1.90) to C-11 (*δ*_C_ 66.7), H-10b (*δ*_H_ 1.69) to C-9 (*δ*_C_ 102.0). This information connected the two partial structures C-2 to C-7 and C-10 to C-16 and thus established the planar structure for the substituted 16-carbon polyketide chain. Comparison of ^13^C chemical shift values for C-5 (*δ*_C_ 70.8) and C-9 (*δ*_C_ 102.0) with that of looekeyolides A and B, and the degrees of unsaturation calculated from the molecular formula suggested the presence of a pyrano ring system within the 16-carbon polyketide acid chain. HMBC correlation between the H-13 of 16-carbon polyketide chain and C-22 carbonyl of phenylalanine, and H-32 (*δ*_H_ 4.86) of 2-deamino-2 hydroxymethionine and C-1 (*δ*_C_ 173.4) carbonyl of 16-carbon polyketide chain were not observed due to paucity of material available for the experiment. The macrocyclic ring system was established in relation to looekeyolides (**1**, **2**) and thus satisfied the molecular formula. These data establish the planar macrocyclic structure for looekeyolide D (**4**). The 2D NOE data and coupling constants for the chiral centers (Table 1) of **4** observed in CD_3_OD were compared with the reported data for **1** in CD_3_OD and the data were comparable. The key NOE data observed between H-4 and H-17, H-5 and H-18, H-7 and H-20, H-10a and H-20, H-10a and H-21, H-13 and H-21, and H-11 and H14 and the comparable coupling constants observed for chiral centers H-3 (10.3, 4.1 Hz), H-5 (11.0, 11.0, 2.0 Hz), H-7 (12.0, 4.8 Hz), 11-H (11.0, 1.7, 1.0 Hz), H-12 (7.0, 1.0, 1.0 Hz) and H-13 (9.6, 6.1, 1.0 Hz) suggested the same relative stereochemistry for the chiral centers in substituted 16-carbon polyketide moiety in **4**.

Next, the absolute configuration of the amino acid phenylalanine and the hydroxmethionine sulfoxide unit was determined. Compound **4** (0.1 mg) was dissolved in EtOH (0.1 mL) and treated with an excess of fresh Raney-Ni (2400) as a slurry in H_2_O (0.1 mL) and refluxed for 0.5 h. The product was filtered and concentrated to give a white solid. Desulfurization with Raney-Ni gave *des*-thiomethyllooekeyolide D. The The desulfurized *des*-thiomethyl product [HRESI/APCIMS *m*/*z* 674.3539 [M + Na]^+^ (calcd for C_34_H_53_NO_11_Na, 674.3511)] was suspended in 6 N HCl (0.1 mL) and heated at 115 °C for 14 h in a sealed tube. The hydrolysate was concentrated to dryness. The residue was reconstituted in 0.1 mL of H_2_O and analyzed by enantioselective HPLC, comparing the retention times with those of authentic standards [Phenomenex Chirex (D) Penicillamine, 4.6 mm × 250 mm, 5 μm]; detection at 254 nm. Using the solvent mixture of 2.0 mM CuSO_4_-MeCN (90:10), with a flow rate of 1.0 mL/min, the retention times (*t*_R_ min) for authentic standards were L-Phe (70.4) and D-Phe (73.3). The *t*_R_ min of the amino acid in the hydrolysate under the same condition was 73.3, indicating the presence of D-Phe in the hydrolysate. The stereochemistry of the α-hydroxy acid was determined using a different chiral column for the HPLC analysis [CHIRALPAK MA (+) (4.6 mm × 50 mm), Diacel Chemical industries, Ltd.; solvent, 2.0 mM CuSO_4_-MeCN (95:5); flow rate, 1.0 mL/min; detection at 254 nm]. The *t*_R_ min for authentic standards were *R*-Hba (9.3) and *S*-Hba (13.3). The retention time of the α-hydroxy acid in the hydrolysate under these conditions was (13.3), indicating the presence of *S*-Hba in the hydrolysate. These data established the absolute configurations for the phenylalanine and hydroxy acid in looekeyolide D (**4**). Looekeyolide D (**4**) is the auto-oxidized product of the 2-deamino-2-hydroxy methionine in looekeyolide C (**3**), and therefore looekeyolide C (**3**) and looekeyoloide D (**4**) have the same chiral centers, thus establishing their structures. HRMS data and stereochemical studies are described in Supplementary Materials.

### 2.3. Bacterial Composition of Belize Samples

**Figure 2:**
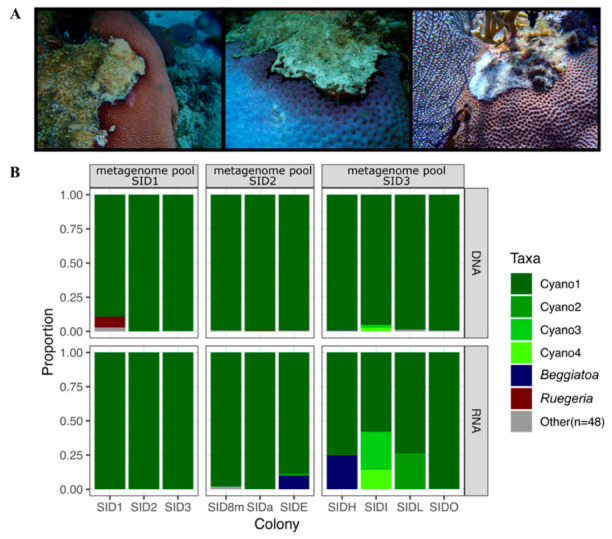
Characterization of black band disease cyanobacterial mats from *Siderastrea siderea* corals. (**A**) Representative photographs of *S. siderea* colonies with black band disease cyanobacterial mats in Belize and Florida. (**B**) Proportions of the V6 region of the 16S rRNA gene amplicon sequence variants in the DNA and RNA fractions of ten cyanobacterial mats from *S. siderea* colonies with black band disease. The predominant amplicon sequence variant called “Cyano1” belongs to the genus *Roseofilum*. Samples were subsequently pooled to ensure sufficient DNA for metagenomic sequencing, as indicated by the clustering labels on the top of the figure.

Bacterial community composition was characterized for both the RNA and DNA fractions of ten Belize *S. siderea* colonies with black band disease (BBD). A total of 1,815,043 16S rRNA gene amplicon sequencing reads passed quality-filtering, with an average of 90,752 reads per sample (min 2973, max 295,333) (Table S1). Only 54 amplicon sequence variants (ASVs) were detected in the twenty libraries (10 RNA, 10 DNA), with just six prevalent ASVs (Table 2, Figure 2B).

The most prevalent ASV, hereafter referred to as Cyano1, ranged from 58 to 100% relative abundance per sample and was classified as the cyanobacterial genus *Roseofilum*. This was the only ASV detected in the RNA fraction of SID1, the sample with the highest number of sequencing reads (295,333). Five additional prevalent ASVs had relative abundances of less than 30% per sample. These included three additional cyanobacterial ASVs, one ASV classified as *Ruegeria*, and one ASV that was classified only as Bacteria with the SILVA database but was identified as *Beggiotoa* through a BLASTN search (Table 2). While the Cyano1 ASV was classified as “*Roseofilum* AO1-A” with SILVA, BLASTN searches of these sequences revealed that they share only 95% sequence similarity in the 60-bp V6 region of the 16S rRNA gene with the *Roseofilum* strain AO1-A (KU579397), isolated from the Great Barrier Reef [11], or with *Roseofilum* strain Cy1 (KP689103), isolated from the Florida Reef Tract [12]. Instead, the Cyano1 (*Roseofilum*) ASV was an exact match to several clone library sequences (EF123634, EF123639, EF123644, EF123645, EF123646) that were previously detected in BBD cyanobacterial mats from Caribbean *S. siderea* corals [13].

The Cyano2 (*Geitlerinema*) ASV was an exact match to several clone library sequences (EF110974, EF154084, DQ644020, DQ680351, DQ151461) that were previously detected in BBD cyanobacterial mats from Caribbean *S. siderea* corals [14,15] and to sequences originating from Caribbean stromatolites (EU917946, EU917928, EU917912, EU917848, EU917831, EU917822, EU917819, EU917807) [16]. Two additional cyanobacterial ASVs, Cyano 3 and Cyano 4, were identified as *Hormoscilla* (Table 2). The *Beggiatoa* ASV shared 96.7% sequence similarity with *Beggiotoa* isolated from BBD on *Montipora capitata* corals in Hawaii [17]. *Ruegeria* strains have also previously been detected in BBD studies [18], including BBD cyanobacterial mats from *S. siderea* corals [19].

### 2.4. Metagenome-Assembled Genomes of Cyanobacteria from Belize and Florida

Four of the Belize *S. siderea* colonies (SIDH, SIDI, SIDL, and SIDO) produced detectable levels of looekeyolide C/D by LC-MS, while six of the colonies (SID1, SID2, SID3, SID8m, SIDa, and SIDE) did not. However, the same predominant cyanobacterial ASV (Cyano 1) was found in both DNA and RNA fractions of BBD from all Belize *S. siderea* colonies. The variability in detection of looekeyolides is likely due to the small sample sizes collected and small amounts of chemical extracts. To confirm the genetic potential for the biosynthesis of looekeyolides, we compared metagenome-assembled genomes (MAGs) of cyanobacteria from pooled metagenomes of producer or non-producer samples (as described in the methods) from Belize as well as producer samples from Florida.

Quality-filtered metagenomic sequencing reads ranged from roughly 13 million to 33 million per metagenomic library (Table S2). Seven cyanobacterial MAGs with >90% completeness and <6% contamination were retrieved from all six metagenome libraries (Table S3). *Roseofilum* MAGs were retrieved from all three Belize metagenomes and one Florida metagenome, encompassing both producers and non-producers of looekeyolide C. In addition, cyanobacterial MAGs that do not belong to the genus *Roseofilum* were retrieved from one Belize metagenome and two Florida metagenomes (Table S3). Among the four *Roseofilum* MAGs from *S. siderea* corals, the average nucleotide identity (ANI) of shared genes was >99%, while the ANI of the *Roseofilum* MAGs compared to non-*Roseofilum* cyanobacteria from *S. siderea* corals was too close to the detection limit for accuracy (<75%), suggesting they belong to different genera (Table S4). *Roseofilum* MAGs from *S. siderea* corals had >98% ANI with *Roseofilum* MAGs retrieved from other Caribbean coral species and had >94% ANI with *Roseofilum* MAGs retrieved from Pacific coral species (Table S4). Of the non-*Roseofilum* MAGs from *S. siderea* corals, SID2_20 and SBC9 had >99% ANI of shared genes with each other and with the *Geitlerinema* BBD 1991 MAG from Caribbean *Montastraea cavernosa* [20,21]. All three strains, SID2_20, SBC9, and BBD 1991 were classified as *Geitlerinema* species by GTDBtk. The non-*Roseofilum* MAG SBLK1 was too close to the detection limit for accuracy (<75%) from SID2_20, SBC9, and *Geitlerinema* BBD 1991. SBLK1 was classified to the cyanobacterial family *Spirulinaceae* by GTDB-Tk. The 16S rRNA gene was not detected in the *Spirulinaceae* bacterium SBLK1 for further taxonomic identification. The presence of three distinct cyanobacterial genera within the order *Oscillatoriales* in the MAGs was consistent with the presence of three cyanobacterial genera (*Roseofilum*, *Geitlerinema*, and *Hormoscilla*) in the 16S rRNA amplicon libraries, although not an exact match for all genera. As the *Roseofilum* strains were the most predominant cyanobacteria within BBD mats from Belize and previous studies from both the Caribbean and Pacific [9,22,23], we focused the pangenome analysis primarily on the *Roseofilum* genomes.

### 2.5. Comparative Genomics of Black Band Disease-Associated Cyanobacteria

**Figure 3:**
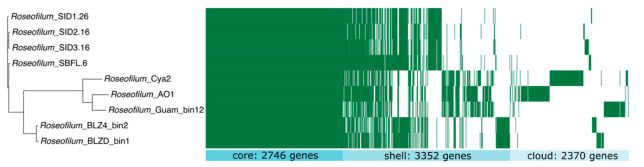
Pangenome comparison of nine *Roseofilum* metagenome-assembled genomes from this study and previously published works. Clustering of genomes is based on the alignment of 2746 core genes present in all nine strains. Shell genes were present in two to eight genomes and cloud genes were present in only one genome.

Comparative genomics of nine *Roseofilum* MAGs included four *S. siderea*-associated *Roseofilum* MAGs from this study and five *Roseofilum* MAGs from other coral species [12,22,23]. All nine MAGs passed the quality threshold of >90% completeness and <6% contamination (Table S3). Pangenome analysis identified 2746 core genes found in all nine *Roseofilum* genomes, 3352 shell genes in two to eight *Roseofilum* genomes, and 2370 cloud genes found in only one *Roseofilum* genome (Figure 3). Three distinct clusters of genomes were detected: Caribbean *Roseofilum* from four *Siderastrea* corals, three Pacific *Roseofilum* strains, and Caribbean *Roseofilum* from two other boulder corals. A total of 294 genes were found in all 4 *Roseofilum* genomes from *S. siderea* but not in any other *Roseofilum* genomes. Of these, 225 (77%) were annotated as hypothetical proteins, while only 69 (23%) had functional annotations.

**Figure 4:**
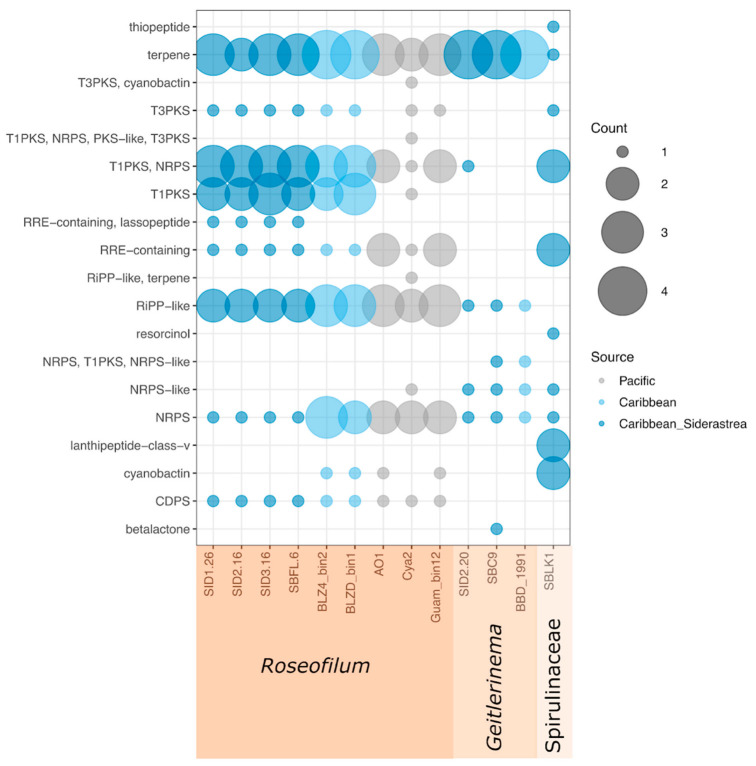
Comparison of biosynthetic gene clusters in thirteen cyanobacterial metagenome-assembled genomes from this study and previously published works. Counts indicate the number of biosynthetic gene clusters rather than individual genes. Looekeyolide and malyngamide clusters are counted as “TIPKS/NRPS”.

Each of the nine *Roseofilum* MAGs had 14 to 19 biosynthetic gene clusters identified by antiSMASH, including multiple clusters for terpenes, ribosomally synthesized and post-translationally modified (RiPP)-like clusters as well as RiPP recognition elements (RRE), Type I polyketide synthases (T1PKSs), nonribosomal peptide synthetases (NRPSs), and hybrid T1PKS/NRPS clusters (Figure 4). Each of the nine *Roseofilum* MAGs had one biosynthetic gene cluster for tRNA-dependent cyclodipeptide synthase (CDPS), which has been more commonly found in the genomes of *Actinobacteria*, *Firmicutes*, and *Proteobacteria* [24,25]. The *Spirulinaceae* MAG also had 14 detectable biosynthetic gene clusters, including gene clusters for antimicrobial lanthipeptides, thiopeptide, and cyanobactins, as well as resorcinol (Figure 4). In contrast to the *Roseofilum* and *Spirulinaceae* MAGs, only eight or nine biosynthetic gene clusters per genome were detected in *Geitlerinema* MAGs.

**Figure 5:**
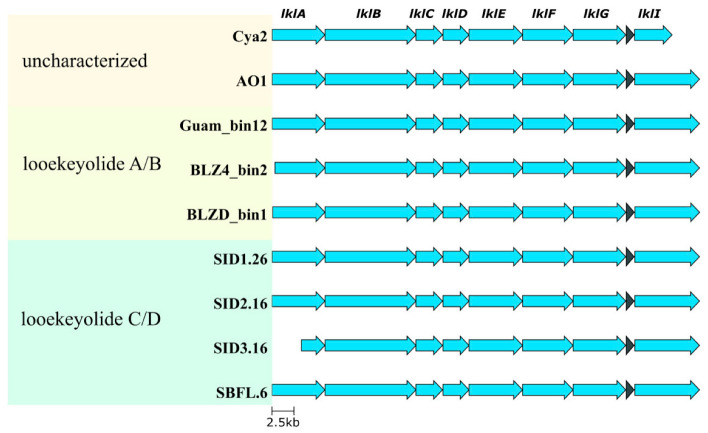
Looekeyolide biosynthetic gene clusters among nine *Roseofilum* metagenome-assembled genomes from this study and previously published works. Natural products have not been characterized from two Pacific *Roseofilum* strains (Cya2, AO1). Looekeyolides A/B have been detected from both Caribbean and Pacific strains (Guam_bin12, BLZ4_bin2, BLZD4_bin1). Looekyolides C/D have only been detected in Caribbean *Roseofilum* from *S. siderea* corals (SID1.26, SID2.16, SID3.16, SBFL.6).

The putative biosynthetic gene clusters for looekeyolides are classified as hybrid T1PKS/NRPS clusters and complete biosynthetic gene clusters were retrieved from all nine *Roseofilum* MAGs (Figure 5), meaning both the producer and non-producer samples had the genetic potential to make looekeyolides, although the level of biosynthesis of the looekeyolides may vary among samples. In addition, two Pacific strains [22,23] appear to have the genetic capacity to produce looekeyolides, but their natural products have not been elucidated.

**Figure 6:**
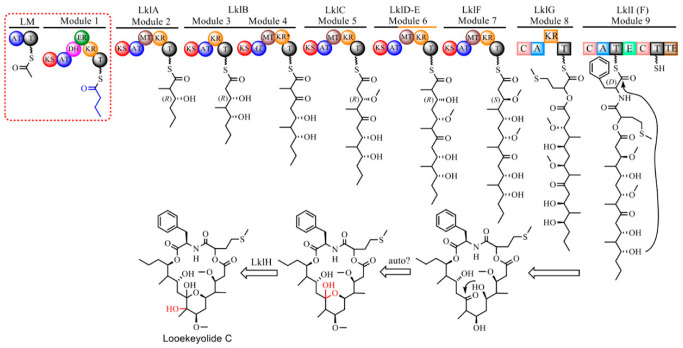
Proposed biosynthetic pathway for looekeyolide C in Caribbean *Roseofilum* metagenome-assembled genomes from *S. siderea* corals.

A putative biosynthetic pathway for looekeyolide C/D is proposed (Figure 6), with high similarity to the pathway for looekeyolide A/B previously described [10] in *Roseofilum* MAGs from corals other than *S. siderea*. The adenylation domain of LklI in *Roseofilum* MAGs from *S. siderea* that produce looekeyolide C/D had specificity for L-phenylalanine, while *Roseofilum* MAGs from corals other than *S. siderea* that produce looekeyolide A/B had specificity for L-leucine (Table S5, Figure S11). Most of the looekeyolide biosynthetic genes in Caribbean *Roseofilum* from multiple coral species (*Orbicella annularis*, *Pseudodiploria strigosa*, *Montastraea cavernosa*) were 97% to 99% similar to the genes in Caribbean *Roseofilum* from *S. siderea* except for LklI which was 92% similar due to the low identity (46%) of their A domains (Figure S12). Hybrid T1PKS/NRPS biosynthetic gene clusters predicted to produce looekeyolides were not detected in *Geitlerinema* or *Spirulinaceae* MAGs.

Each of the nine *Roseofilum* MAGs had hybrid T1PKS/NRPS clusters annotated as malyngamides including malyngamide C acetate and malyngamide I (Figure S13). Malyngamides are small amides, many of which have lyngbic acid as a carboxylic acid side chain. Both malyngamides and lyngbic acid from Caribbean filamentous cyanobacteria, including *Roseofilum*, have previously been shown to interfere with bacterial quorum sensing [9,26]. Most of the nine *Roseofilum* MAGs had multiple putative malyngamide biosynthetic gene clusters, and no clear patterns were observed that corresponded to differences in gene clusters among coral hosts or geographic locations (Figure S13). Hybrid T1PKS/NRPS biosynthetic gene clusters predicted to produce malyngamides were not detected in *Geitlerinema* or *Spirulinaceae* MAGs.

**Figure 7:**
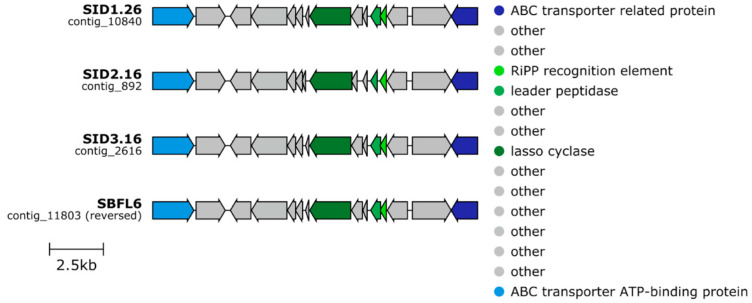
Lasso peptide biosynthetic gene clusters detected only in Caribbean *Roseofilum* metagenome-assembled genomes from *S. siderea* corals.

Analysis of biosynthetic gene clusters also revealed that the *Roseofilum* MAGs from *S. siderea* corals had one type of biosynthetic gene cluster that was not found in the other *Roseofilum* strains. All four *Roseofilum* MAGs from *S. siderea* corals had a lasso peptide biosynthetic gene cluster that encoded a 98 aa stand-alone RiPP recognition element (RRE), a 135 aa lasso peptide transglutaminase homolog (leader peptidase, capB), and a 633 aa lasso peptide asparagine synthase homolog (lasso cyclase, capC). These biosynthetic genes were flanked on each side by genes for ABC-transporter related genes (Figure 7). While the amino acid sequences in the RiPP recognition element and the capB leader peptidase were identical in all four *Roseofilum* MAGs from *S. siderea* corals, the amino acid sequences for the capC lasso cyclase in SID1.26 and SBFL6 differed by 4 amino acids from SID2.16 and SID3.16. A search with blastp of the *S. siderea*-associated *Roseofilum* lasso cyclase amino acid sequence (from SID2.16) showed low similarity (≤67% similarity) to homologues in other cyanobacterial genomes.

## 3. Discussion

*Roseofilum reptotaenium*, the cyanobacterial engineer of black band disease (BBD) in corals [7], is found in tropical coral reefs around the world and impacts at least 72 coral species [27]. Here, we uncovered cryptic diversity among *Roseofilum* strains through both chemical and genomic analyses. The sequence-based threshold of 95% ANI has been proposed as the delineation of bacterial species [28,29,30,31]. Using this metric, all six *Roseofilum* strains from the Caribbean are the same cyanobacterial species regardless of the host coral species (>98% ANI), while the three Pacific *Roseofilum* strains were very close to this threshold (94.25–94.68% ANI) and thus, potentially represent a separate species. However, using ANI for comparison only reveals the similarity among shared genes and does not capture differences in gene content, i.e., when genes are present in one strain and absent in another. *Roseofilum* strains on *S. siderea* corals were both chemically and genetically distinct from other strains in the Caribbean despite belonging to the same cyanobacterial species. This difference was consistent across sites in Belize and Florida and through time, as samples were collected in 2014, 2015, and 2018. Of note, surveys using only 16S rRNA amplicons would not be able to distinguish among these distinct strains of *Roseofilum*, thus highlighting the utility of metagenomic sequencing in uncovering the functional differences among visually similar filamentous cyanobacteria in reef ecosystems.

Over 2000 metabolites have been described from *Cyanobacteria* [32]. Some of the ecological roles of these natural products include grazing deterrents, allelopathy, iron scavenging, UV protection, and signaling [33]. Comparative analysis of nine *Roseofilum* genomes showed that each of the *Roseofilum* genomes had multiple terpene, ribosomal and non-ribosomal peptide, and polyketide biosynthetic gene clusters. Each of these classes of cyanobacterial natural products includes potential antibacterial or antiviral compounds [34,35,36]. In fact, every type of biosynthetic cluster detected in these BBD-associated cyanobacterial genomes, regardless of genus, includes natural products that exhibit antimicrobial properties. These antimicrobial agents may play a role in the progression of BBD by allowing the cyanobacteria to outcompete other coral-associated microorganisms that would normally suppress pathogen growth.

All *Roseofilum* genomes examined here had hybrid peptide/polyketide biosynthetic gene clusters proposed to encode for the cyclic depsipeptide looekeyolides, the lipopeptide malyngamides, and a tRNA-dependent cyclodipeptide that were not found in four non-*Roseofilum* BBD-associated cyanobacterial genomes. Our previous work demonstrated that looekeyolides from *Roseofilum* under laboratory conditions do not alter growth and biofilm formation by marine bacteria, do not act as siderophores, and do not impact photosynthetic performance of the coral [10]. The oxygen sensitive looekeyolide A reduces hydrogen peroxide levels, suggesting a role in combating reactive oxygen species on the coral surface [10]. Malyngamides from filamentous cyanobacteria have demonstrated both cytotoxic and anticancer properties [37] and antibacterial properties against Gram positive pathogens [38]. Malyngamide C and lyngbic acid have also demonstrated quorum-sensing inhibition in marine bacteria [9,26]. In addition, the tRNA-dependent cyclodipeptides have variously shown antibacterial, antifungal, antiviral, and antitumor properties [39]. In contrast to looekeyolides and malyngamides, only the *S. siderea*-associated *Roseofilum* genomes contained biosynthetic gene clusters for lasso peptides. Lasso peptides are underexplored in Cyanobacteria [40]. Characterized lasso peptides have demonstrated a variety of activities including antimicrobial properties, and the unique lasso structure imparts heat and chemical resistance [41].

The biosynthesis of malyngamides and lasso peptides has been well characterized [42,43], setting the stage for their heterologous production and bioactivity investigation. In addition to the proposed biosynthetic pathway for looekeyolide C presented here, we recently proposed a pathway for looekeyolide A [10]. With a cultivated strain of *Roseofilum* that produces looekeyolide A [10] and the genome sequences for multiple, unique *Roseofilum* strains, we are poised for future studies to uncover the bioactivity of these natural products and their potential use for novel applications.

Collectively, *Roseofilum* genomes associated with BBD from locations in the Caribbean and the Pacific share a wide assortment of peptide and polyketide natural products that may have bioactive properties. The exact roles of looekeyolides, malyngamides, and other secondary metabolites are not known, but the conserved nature of these compounds implies they play an important role in the ecology of these cyanobacteria and may also contribute to disease etiology through manipulation of the microbial communities around them.

## 4. Materials and Methods

### 4.1. Sample Collection and Enrichment Culturing

Black band disease (BBD) cyanobacterial mats were collected from *Siderastrea siderea* corals in Belize and Florida by aspiration with a needleless syringe for both chemical analysis and extraction of nucleic acids. BBD mats from several colonies of *Siderastrea* were combined for bulk analysis in three batches: one from South Water Caye, Belize in July 2014, one from Curlew Cay, Belize in August 2018, and one from Fort Lauderdale, Florida in July 2018. For microbiome analysis, relatively thin BBD mats (Figure 1) from ten colonies of *S. siderea* were sampled while SCUBA diving in September 2015 at Carrie Bow Cay, Curlew Cay, or South Water Channel near the Smithsonian Carrie Bow Cay Field Station in Belize. One additional *S. siderea* coral exhibiting BBD was sampled at Looe Key in the Florida Keys National Marine Sanctuary in July 2017. Finally, a BBD mat was collected from a *S. siderea* coral offshore from Ft. Lauderdale, FL in July 2018. A non-axenic, cyanobacterial enrichment culture of the BBD mat from Ft. Lauderdale, FL was grown in artificial seawater amended with Cyanobacterial BG-11 media (ATCC medium 616) as previously described [12].

### 4.2. Characterization of Major Secondary Metabolites

Bulk cyanobacterial mats of the 2014 collection were freeze-dried and extracted repeatedly with MeOH. Similarly, the 2018 collection was freeze-dried and extracted with 50% EtOAc-50% MeOH saturated with helium gas. The extracts were chromatographed on a column of C_18_ (3 g) using a MeOH-H_2_O step gradient system to give five sub-fractions. The sub-fraction 3 (0.002 g), eluted with 80% MeOH-20% H_2_O was further separated by reversed-phase HPLC (semi-prep 250 mm × 10 mm, 5 μm, RP-18, flow 3.0 mL/min) using 80% MeOH-20% H_2_O to give 0.6 mg of looekeyolide D (*t*_R_ = 10.3 min, yield, 0.03% dry wt) (July 2014 batch) and 0.3 mg of looekeyolide D (*t*_R_ = 10.3 min, yield, 0.06% dry wt) (August 2018 batch). Looekeyolide C was not isolated and assumed to be completely oxidized during the isolation process.

Optical rotations were recorded on a Jasco P2000 polarimeter. UV spectrophotometric data was acquired on a Shimadzu PharmaSpec UV-visible spectrophotometer. NMR data were collected on a JEOL ECA-600 spectrometer operating at 600.17 MHz for ^1^H and 150.9 MHz for ^13^C. ^1^H NMR chemical shifts (referenced to residual CD_3_OD at *δ* 3.30) were assigned using a combination of data from 2D DQF COSY and multiplicity-edited HSQC experiments. The edited-HSQC experiment was optimized for *J*_CH_ = 140 Hz and the HMBC experiment was optimized for ^2/3^*J*_CH_ = 8 Hz. ^13^C NMR chemical shifts (referenced to CD_3_OD observed at *δ* 49.0) were assigned on the basis of multiplicity-edited HSQC experiments. Low resolution liquid chromatography mass spectrometry (LRLC-MS) was performed on a Thermo Scientific (Waltham, MA, USA) LTQ LC-MS ESI instrument connected to a Grace Vydac Reversed-phase column (C18, 218TP, 5 μ, 100 mm × 2.1 mm) using a mixture of 0.1% HCOOH in water (A) and 0.1% HCOOH in CH_3_CN (B) at a rate of 0.2 mL/min. The gradient system used was 90% A to 0% A in 15 min followed by 100% B for the next 10 min. HRMS data was obtained using an Agilent 6210 LC-TOF mass spectrometer equipped with an APCI/ESI multimode ion source detector at the Mass Spectrometer Facility at the University of California, Riverside, California. Varian BondElut octadecyl (C_18_) was used for column chromatography. All solvents used were of HPLC grade (Fisher Scientific).

### 4.3. Nucleic Acids Extraction

Seawater was decanted from the self-clumping BBD mats and mats were either frozen immediately (Ft. Lauderdale sample) or preserved (Belize and Looe Key samples) with 5–10 volumes of RNAlater (Qiagen, Germantown, MD, USA) before freezing at −80 °C. DNA and RNA were co-extracted with an Allprep DNA/RNA mini kit (Qiagen, Germantown, MD, USA) from the Belize samples. RNA was treated with DNase I (New England Biolabs, Ipswich, MA, USA), concentrated with RNA Clean & Concentrate (Zymo Research, Irvine, CA, USA), and cDNA was synthesized with a VILO Superscript cDNA Synthesis kit (Invitrogen, Carlsbad, CA, USA). DNA was extracted from the two Florida field samples and from the cyanobacterial enrichment culture with a Dneasy Powersoil DNA extraction kit (Qiagen, Germantown, MD, USA) according to the manufacturer’s instructions with the addition of 12 μL of proteinase K (New England Biolabs, Ipswich, MA, USA) to the bead tube with solution C1 and incubated 30 min at room temperature before the bead beating step.

### 4.4. V6 Amplicon Libraries of Belize Samples

The V6 region of bacterial 16S rRNA genes were amplified from both DNA and cDNA of the Belize samples with previously published primers [44] using previously described methods [9]. Briefly, the V6 region was amplified in triplicate with Phusion High-Fidelity Polymerase (New England Biolabs, Ipswich, MA, USA). Triplicate PCR amplifications were pooled for each sample, cleaned with a MinElute kit (Qiagen, Germantown, MD, USA), and quantified by NanoDrop (ThermoScientific, NanoDrop Products, Wilmington, DE, USA). Two hundred nanograms of each cleaned amplicon library was submitted to the Interdisciplinary Center for Biotechnology Research at the University of Florida (RRID:SCR_019152) where the libraries were size selected for fragments from 200 to 240 bp with a 2% agarose PippinPrep cassette and cleaned again to remove agarose. Sequencing was performed on an Illumina MiSeq with a 150-bp paired-end protocol, using single indexing. Sequencing reads were parsed by Illumina index at the sequencing center and further parsed by the inline barcode using with the command-line options of FASTX-toolkit (http://hannonlab.cshl.edu/fastx_toolkit/ (accessed on 30 July 2018)). Primers and adaptors were removed using cutadapt v. 2.8 [45] and sickle v. 1.33 [46]. Parsed, quality-filtered amplicon sequencing reads are publicly available through NCBI’s Sequence Read Archive under the Bioproject ID PRJNA645365. Quality-filtered paired reads were merged and amplicon sequence variants were determined from de-replicated sequences using taxonomic assignment from the SILVA small subunit ribosomal RNA database v. 132 database [47] with DADA2 v. 1.10.1 [48]. Sequences classified as mitochondria or chloroplast were removed from further analysis. Prevalent sequences that were unclassified were searched against NCBI’s non-redundant nucleotide collection with BLASTn [49]. Bacterial community analysis was completed with phyloseq v. 1.26.1 [50] and plotted with ggplot2 v. 3.1.1 [51].

### 4.5. Metagenomic Library Preparation

A total of six metagenomic libraries were prepared. To ensure enough DNA for library preparation, extracted DNA from Belize samples were pooled as follows. Samples SID1, SID2, and SID3 were pooled for metagenome library “SID1”, samples SID8m, SIDa, and SIDE were pooled for metagenome library “SID2”, and samples SIDH, SIDI, SIDL, and SIDO, all known producers of looekeyolide C/D, were pooled for metagenome library “SID3”. The three pooled DNA samples from Belize were sent to the University of Maryland Institute for Bioscience and Biotechnology Research where metagenomic libraries were prepared with a TruSeq DNA Sample Preparation Kit (Illumina, San Diego, CA, USA) and sequenced on an Illumina HiSeq with a 100-bp paired-end protocol. Metagenomic libraries for the three Florida samples were prepared with a Nextera DNA Flex kit (Illumina, San Diego, CA, USA) and sequenced on an Illumina NextSeq500 at the University of Florida Interdisciplinary Center for Biotechnology Research with a 150-bp paired-end protocol.

### 4.6. Metagenomic Analysis

Quality-filtering and removal of sequencing adaptors of the 100-bp sequencing reads of the Belize samples was performed with cutadapt v. 2.8 [45] and sickle v. 1.33 [46] with a removal of all reads with Ns, a minimum quality score of 30, and a minimum length of 100 bp. Quality-filtering of the 150-bp sequencing reads of the Florida samples was performed with the Minoche [52] filtering pipeline in illumina-utils v. 2.3 [44] and sequencing adaptors were removed with cutadapt v. 2.8 and sickle v. 1.33. Quality-filtered and adaptor-free metagenomic sequencing reads are publicly available through NCBI’s Sequence Read Archive under the Bioproject ID PRJNA647383. Metagenomic libraries for Belize samples were assembled with MetaSPAdes v. 3.12 [53] and metagenomic libraries for Florida samples were assembled with MEGAHIT v. 1.1.4 [54,55]. Unassembled quality-filtered sequencing reads were mapped to the metagenomic assemblies with bowtie2 v. 2.3.5.1 [56] and sorted with SAMtools v. 1.10 [57].

Metagenome-assembled genomes (MAGs) were retrieved by binning of contigs with MetaBAT v. 2.13 [58]. Cyanobacterial MAGs from this study as well as our previously published BBD cyanobacterial MAGs [12] are publicly available through NCBI’s Sequence Read Archive under the Bioproject ID PRJNA647383. Genome quality was assessed with the Microbial Genomes Atlas (MiGA) online [59]. Taxonomic classification of cyanobacterial MAGs was performed with GTDB-Tk v. 2.1.0 and database version R207_v2 using default settings [60,61]. The average nucleotide identity of shared genes was assessed pairwise with the Average Nucleotide Identity calculator from the enveomics toolbox [62]. The genomes of closely related strains of *Roseofilum*, including four strains from this study and five previously published strains [12,22,23], were annotated with Prokka v. 1.12 [63] and comparative genomic content was analyzed with Roary v. 3.12.0 [64]. An approximately-maximum-likelihood phylogenetic tree of the nine *Roseofilum* genomes was created from the alignment of core genes with FastTree v. 2.1.7 [65] and plotted with Phadango v. 1.3.0 [66]. Biosynthetic gene clusters were identified with the online antiSMASH database bacterial version 6 [67] and with PRISM4 v. 4.4.5 [68]. Biosynthetic gene clusters were visualized with clinker v. 0.0.21 [69] and edited with inkscape v. 1.1.0 [70,71,72].

## Figures and Tables

**Table 1:** NMR spectroscopic data for looekeyolide D (**4**) in CD_3_OD (600 MHz).

Position	*δ*_C_ Mult.	*δ*_H_ (*J* in Hz)	COSY *^a^*	HMBC	NOESY *^b^*
1	173.4, C			2a	
2a	33.6, CH_2_	2.52, d (12.0)	2b,		2b, 3, 5
2b		2.36, dd (12.0, 10.3)	2a, 3		2a, 3
3	77.6, CH	4.34, dd (10.3, 4.1)	2b, 4	2, 18	2a, 2b, 4, 11
4	38.7, CH	2.21, m	3, 5, 18	6b, 18	3, 17, 18
5	70.8, CH	3.60, ddd (11.0, 11.0, 2.0)	4, 6a, 6b	6b, 18	2a, 6a, 18
6a	31.4, CH_2_	2.08, ddd (12.1, 11.0, 4.8)	5, 6b, 7		5, 6b, 7, 18
6b		1.42, ddd (12.1, 12.0, 1.8)	5, 6a,		6a
7	80.0, CH	3.44, dd (12.0, 4.8)	6a, 6b	6a, 6b, 20	6a, 10a, 20
8	74.7, C			6a, 6b, 20	
9	102.0, C			10b, 20	
10a	37.9, CH_2_	1.90, ddd (12.3, 11.0)	10b, 11		7, 10b, 11, 20, 21
10b		1.69, dd (12.3, 2.7)	10a, 11		10a, 11, 12
11	66.7, CH	4.70, ddd (11.0, 1.7, 1.0)	10a, 10b, 12	10a, 12, 21	3, 10ab, 14a, 14b
12	42.6, CH	1.52, ddq (7.1, 1.0, 1.0)	11, 13, 21	21	10b, 13, 21
13	81.4, CH	4.99, ddd, (9.6, 6.1, 1.0)	12, 14a, 14b	21	12, 21
14a	35.4, CH_2_	2.15, m	13, 14b, 15a, 15b	12, 16	14b, 15b
14b		1.59, m	13, 14a, 15a, 15b	14a	
15a	20.3, CH_2_	1.36, m	14a, 14b, 15b, 16	16	15b, 16
15b		1.29, m	14a, 14b, 15a, 16		15a, 16
16	14.3, CH_3_	0.93, t (7.5)	15a, 15b		15a, 15b
17	57.4, OCH_3_	3.32, s			4
18	9.7, CH_3_	0.81, d (6.9)	4		4, 5, 6a
19	57.7, OCH_3_	3.38, s			20
20	19.6, CH_3_	1.25, s		7	7, 10a, 19
21	11.9, CH_3_	0.98, d (6.8)	12		12, 13, 10a
22	175.1, C				
23	53.6, CH	4.81, dd (10.8, 4.2)	24a, 24b	24a, 24b	
24a	36.0, CH_2_	3.39, m	23, 24b	26, 30	24b
24b		2.82, m	23, 24a		24a
25	138.9, C			27, 29	
26	130.0, CH	7.23, d (6.8)	27	28	27
27	129.7, CH	7.27, t (6.8)	26, 28		26, 28
28	127.9, CH	7.19, t (6.8)	27, 29	26, 30	27, 29
29	129.7, CH	7.27, t (6.8)	28, 30		28, 30
30	130.0, CH	7.23, d (6.8)	29	28	29
31	171.7, C				
32	73.6, CH	4.86, dd (8.9, 4.0)	33	33	33a, 33b
33	25.4, CH_2_	1.83, m	32, 34a, 34b	32, 34	32, 34
34a	48.9, CH_2_	2.58, m	33, 34b	33, 35	34b
34b		2.46, m	33, 34a		34a
35	37.3, CH_3_	2.508, 2.501, s		34	

*^a^* COSY and NOESY correlations are from proton(s) stated to the indicated protons. *^b^* HMBC correlations are from proton(s) stated to the indicated carbons.

**Table 2:** Six predominant Amplicon Sequence Variants (ASVs) in V6 amplicon libraries from black band disease (BBD) cyanobacterial mats from *S. siderea* corals in Belize. For each ASV, the SILVA classification is provided, as well as the closest BLAST match in GenBank for comparison. Accession numbers in bold indicate sequences originating from previous BBD studies.

ASV Name	SILVA Classification	Closest BLAST Match (% Similarity for V6 Region)
Cyano 1	*Roseofilum* AO1-A	**EF123646** (100%)
Cyano 2	*Geitlerinema* PCC-7105	**EF110974** (100%), **EF372580** (100%)
Cyano 3	*Hormoscilla* SI04-45	KY697267 (100%)
Cyano 4	*Hormoscilla* SI04-45	KY697265 (100%)
*Beggiatoa*	unclassified genus of Bacteria	**KM924160** (96.7%)
*Ruegeria*	*Ruegeria*	MT484146 (100%)

## Data Availability

Metagenomic sequencing reads and metagenome-assembled genomes are available in GenBank under Bioproject PRJNA647383. Amplicon sequencing reads are available in GenBank under Bioproject PRJNA645365.

## Supplementary Materials

The following supporting information can be downloaded at: https://www.mdpi.com/article/10.3390/md21020076/s1, Figure S1: LRLC-MS data showing the presence of looekeyolides C and D in the MeOH extract of 2014 Belize collection; Figure S2: LRLC-MS data showing the presence of looekeyolides C and D in the EtOAc-MeOH (1:1) extract of 2018 Belize collection; Figure S3: LRLC-MS data showing the presence of looekeyolides C and D in the EtOAc-MeOH (1:1) extract of 2018 Fort Lauderdale, Florida collection; Figure S4: LRLC-MS data showing the presence of looekeyolides C and D in fraction 3 of the 2014 Belize collection; Figure S5: ^1^H NMR (600 MHz, CD_3_OD) spectrum of looekeyolide D (**4**); Figure S6: ^13^C NMR (151 MHz, CD_3_OD) spectrum of looekeyolide D (**4**); Figure S7: DQF-COSY NMR (600 MHz, CD_3_OD) spectrum of looekeyolide D (**4**); Figure S8: HSQC NMR (600 MHz, CD_3_OD) spectrum of looekeyolide D (**4**); Figure S9: HMBC NMR (600 MHz, CD_3_OD) spectrum of looekeyolide D (**4**); Figure S10: 2D-NOESY NMR (600 MHz, CD_3_OD) spectrum of looekeyolide D (**4**); Figure S11: Alignment of A domains of LklI and LklI(F) by ClustalW; Figure S12: Comparison of gene similarities in the biosynthetic gene clusters for looekeyolide A/B and looekeyolide C/D. Figure S13: Biosynthetic gene clusters for malyngamides in *Roseofilum* MAGs; Table S1: Metadata and sequencing read metrics for V6 amplicon libraries from Black Band Disease cyanobacterial mats from *Siderastrea siderea* corals in Belize; Table S2: Metadata and sequencing read metrics for metagenomic libraries from Black Band Disease cyanobacterial mats from *Siderastrea siderea* corals in Belize and Florida; Table S3: Quality metrics of metagenome-assembled genomes (MAGs) of (A) non-*Roseofilum* cyanobacteria from *Siderastrea siderea*, (B) *Roseofilum* from *Siderastrea siderea*, and C) previously published *Roseofilum* strains; Table S4: (A) Pairwise Average Nucleotide Identity (ANI) of shared genes among cyanobacterial MAGs from Black Band Disease on *Siderastrea siderea* corals. Values of 75% or below are too close to the detection limit for confident assessments. Values above this threshold are highlighted in green, (B) Pairwise Average Nucleotide Identity (ANI) of shared genes among *Roseofilum* MAGs from Black Band Disease on multiple coral species. Table S5: Specificity codes for adenylation (A) domains of two NRPSs for the biosynthesis of Lk-A/B and Lk-C/D.
