# Supplementary material for: Cryptic Diversity of Black Band Disease Cyanobacteria in Siderastrea siderea Corals Revealed by Chemical Ecology and Comparative Genome-Resolved Metagenomics
Source: Mar Drugs. 2023 Jan 22;21(2):76. doi: 10.3390/md21020076 (PMC9967302; doi:10.3390/md21020076)
Supplement: Supplementary file 1 [file marinedrugs-21-00076-s001.zip › marinedrugs-2120723-supplementary.pdf]

Supplementary Materials for "Cryptic Diversity of Black Band Disease Cyanobacteria in Siderastrea siderea Corals Revealed by Chemical Ecology and Comparative Genome-Resolved Metagenomics"

Julie L. Meyer 1,*, Sarath P. Gunasekera 2, Anya L. Brown 3,4, Yousong Ding 5, Stephanie Miller 1, Max Teplitski 1,2 and Valerie J. Paul 2

1 Department of Soil, Water, and Ecosystem Sciences, University of Florida, Gainesville, FL 32610, USA

2 Smithsonian Marine Station, Ft. Pierce, FL 34949, USA

3 School of Natural Resources and Environment, University of Florida, Gainesville, FL 32603, USA;

4 Department of Evolution and Ecology & Bodega Marine Lab, University of California,

Bodega Bay, CA 94923, USA

5 Department of Medicinal Chemistry & Center for Natural Products, Drug Discovery and Development, University of Florida, Gainesville, FL 32603, USA

* Correspondence: juliemeyer@ufl.edu

General Experimental Procedures.

The optical rotations were recorded on a Jasco P2000 polarimeter. UV spectrophotometric data was acquired on a Shimadzu PharmaSpec UV-visible spectrophotometer. NMR data were collected on a JEOL ECA-600 spectrometer operating at 600.17 MHz for ^{1}H and 150.9 MHz for ^{13}C. ^{1}H NMR chemical shifts (referenced to residual CD_3OD at δ 3.30) were assigned using a combination of data from 2D DQF COSY and multiplicity-edited HSQC experiments. The edited-HSQC experiment was optimized for J_{CH} = 140 Hz and the HMBC experiment was optimized for $^{2/3}J_{CH}$ = 8 Hz. ^{13}C NMR chemical shifts (referenced to CD_3CN observed at δ 118.2 and CD_3OD at δ 49.0) were assigned on the basis of multiplicity-edited HSQC experiments. The LC-MS data were obtained on LC electrospray ionization MS system with a LTQ Advantage Max spectrometer (Thermo Finnigan, Waltham, MA, USA). The HRMS data was obtained using an Agilent 6210 LC-TOF mass spectrometer equipped with an APCI/ESI multimode ion source detector at the Mass Spectrometer Facility at the University of California,

Riverside, California. Varian BondElut octadecyl (C_{18}) were used for column chromatography. All solvents used were of HPLC grade (Fisher Scientific).

Collection, Extraction, and Isolation The cyanobacterial mats from the black-band disease zones from several samples of coral species of *Siderastrea siderea* growing near South Water Cay, Belize were collected on July, 2014 and bulked for this study. This collection was freeze dried to give a dry weight of 1.96 g. This dry material was extracted repeatedly with MeOH. Concentration of the combined extracts by rotary evaporation at 45 ^{o}C under reduced pressure gave 173 mg of the MeOH soluble fraction. The LRESI MS analysis of the MeOH extract as shown in Figure S1 indicated the presence of naturally occurring unstable looekeyolide C (m/z = 720) and traces of the relatively stable oxidized product looekeyolide D (m/z = 736). The MeOH extract (0.173 g) was chromatographed on a column of C_{18} (3 g) using MeOH–H_2O step gradient system to give five sub-fractions. The LRESI MS analysis of the fraction 3, as shown in Figure S4, indicated the presence of less naturally occurring unstable looekeyolide C (m/z = 720) and more of its relatively stable oxidized product looekeyolide D (m/z = 736) indicating the oxidation during the chromatography procedure. The sub-fraction 3 (0.002 g), eluted with MeOH–%02 H_2O was further separated by reversed-phase HPLC (semi-prep 250 x 10 mm, 5 μm, RP-18, flow 3.0 mL/min) using MeOH–%02 H_2O to give 0.6 mg of looekeyolide D (t_R = 10.3 min, yield, 0.03% dry wt). Looekeyolide C was not isolated and assumed to be oxidized during the HPLC separation.

A second batch of cyanobacterial mat from the black-band disease zones of *Siderestrea* growing in Curlew Cay, Belize was collected on August 6, 2018. The freeze-dried material 0.482 g was extracted with EtOAc–%05 MeOH saturated with helium gas. Concentration of the extract as above furnished 0.029 g of EtOAc-MeOH soluble fraction. The LRESI MS analysis of

the EtOAc–MeOH soluble fraction as shown in Figure S2 indicated the presence of relatively equal amounts of naturally occurring unstable looekeyolide C (*m/z* = 720) and its relatively stable oxidized product looekeyolide D (m/z = 736). Reversed-phase C_{18} column chromatography of the extract followed by reversed-phase HPLC under the same conditions gave 0.3 mg of looekeyolide D (t_R = 10.3 min, yield, 0.06% dry wt). The unstable looekeyolide C oxidized to looekeyolide D during the separation procedures.

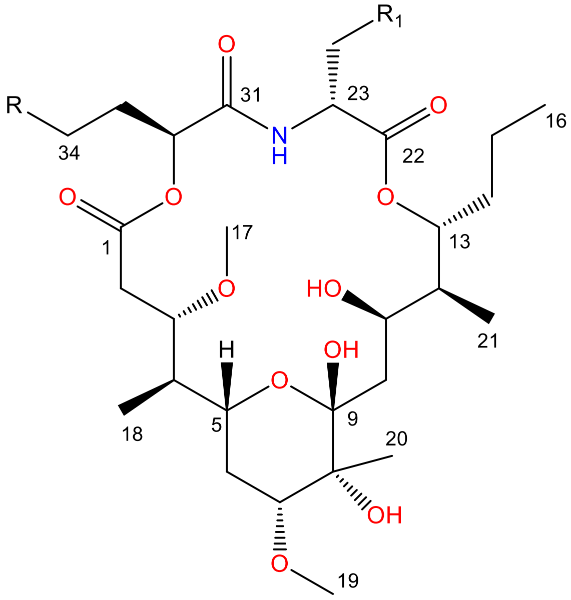

Looekeyolide A (**1**), R = S-CH_3, R_1 = $CH(CH_3)_2$
Looekeyolide B (**2**), R = S(O)-CH_3, R_1 = $CH(CH_3)_2$
Looekeyolide C (**3**), R = S-CH_3, R_1 = Ph
Looekeyolide D (**4**), R = S(O)-CH_3, R_1 = Ph

Gunasekera SP, Meyer JL, Ding Y, Abboud KA, Luo D, Cambell JE, Angerhofer A, Goodsell JL, Raymundo LJ, Lui J, Ye T, Luesch H, Teplitski M, Paul VJ. Chemical and metagenomic studies of the lethal black band disease of corals reveal two broadly distributed, redox-sensitive mixed polyketide/peptide macrocycles. *J. Nat. Prod*. 2019; ***82***: 111–121.

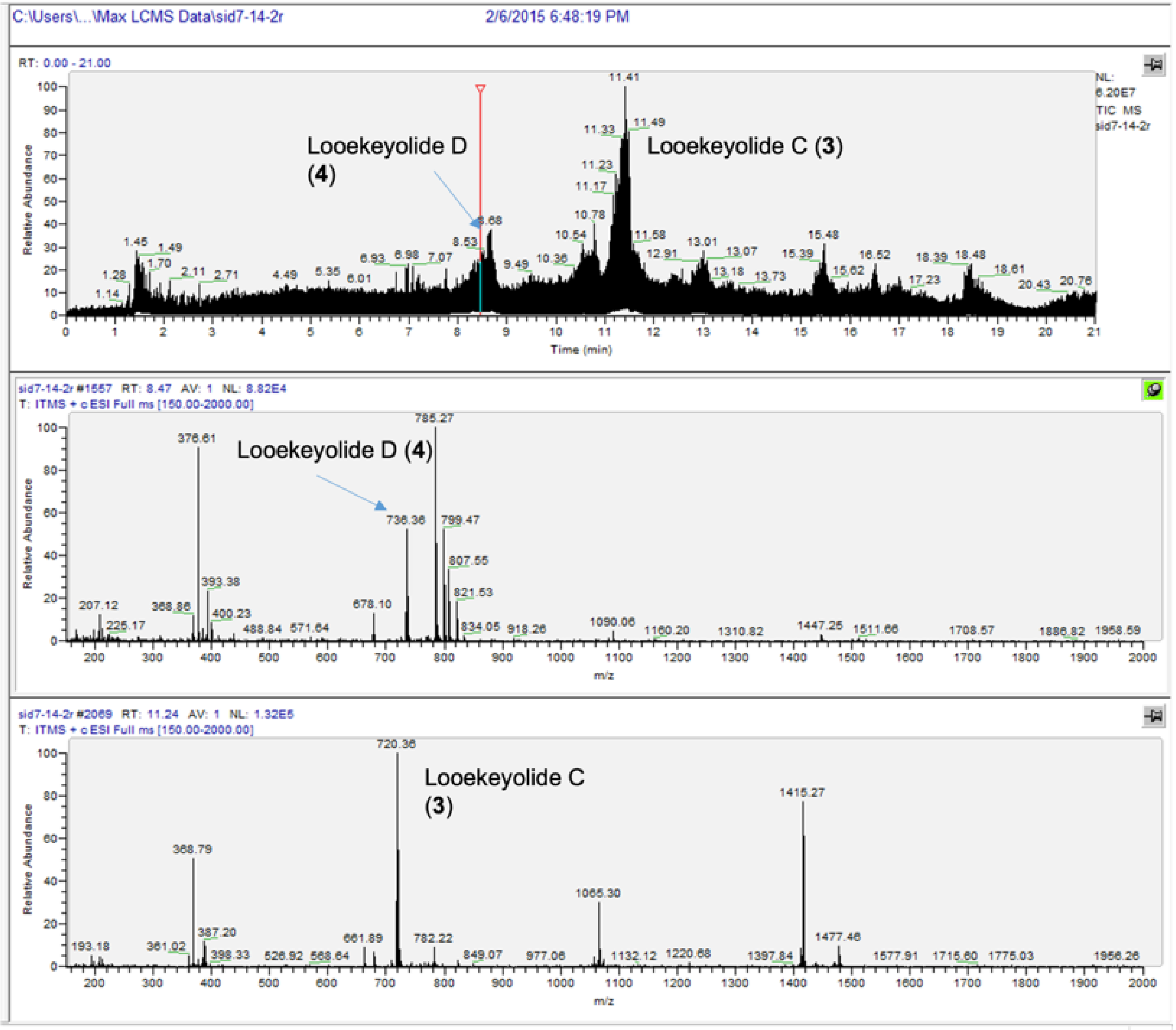

Figure S1: LRLC-MS data showing the presence of looekeyolides C and D in the MeOH extract of 2014 Belize collection.

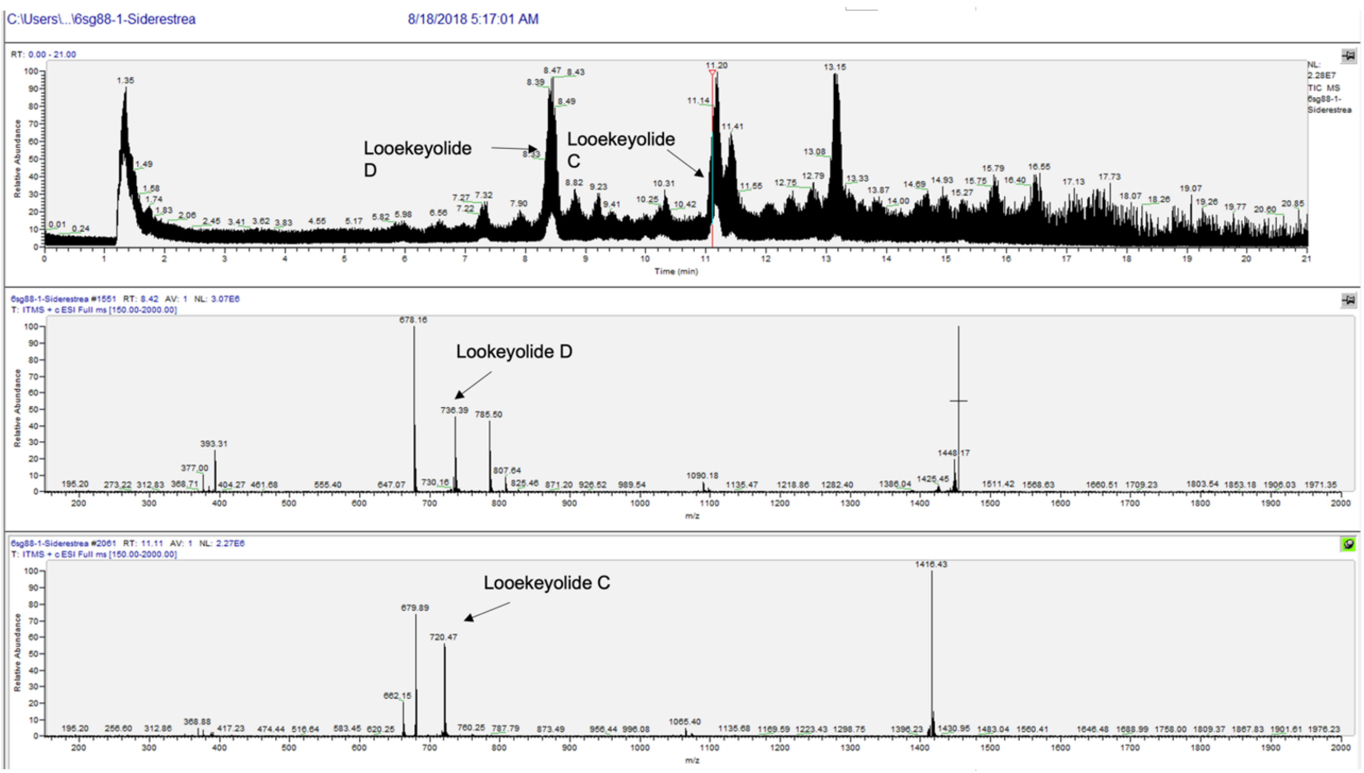

Figure S2: LRLC-MS data showing the presence of looekeyolides C and D in the EtOAc-MeOH (1:1) extract of 2018 Belize collection.

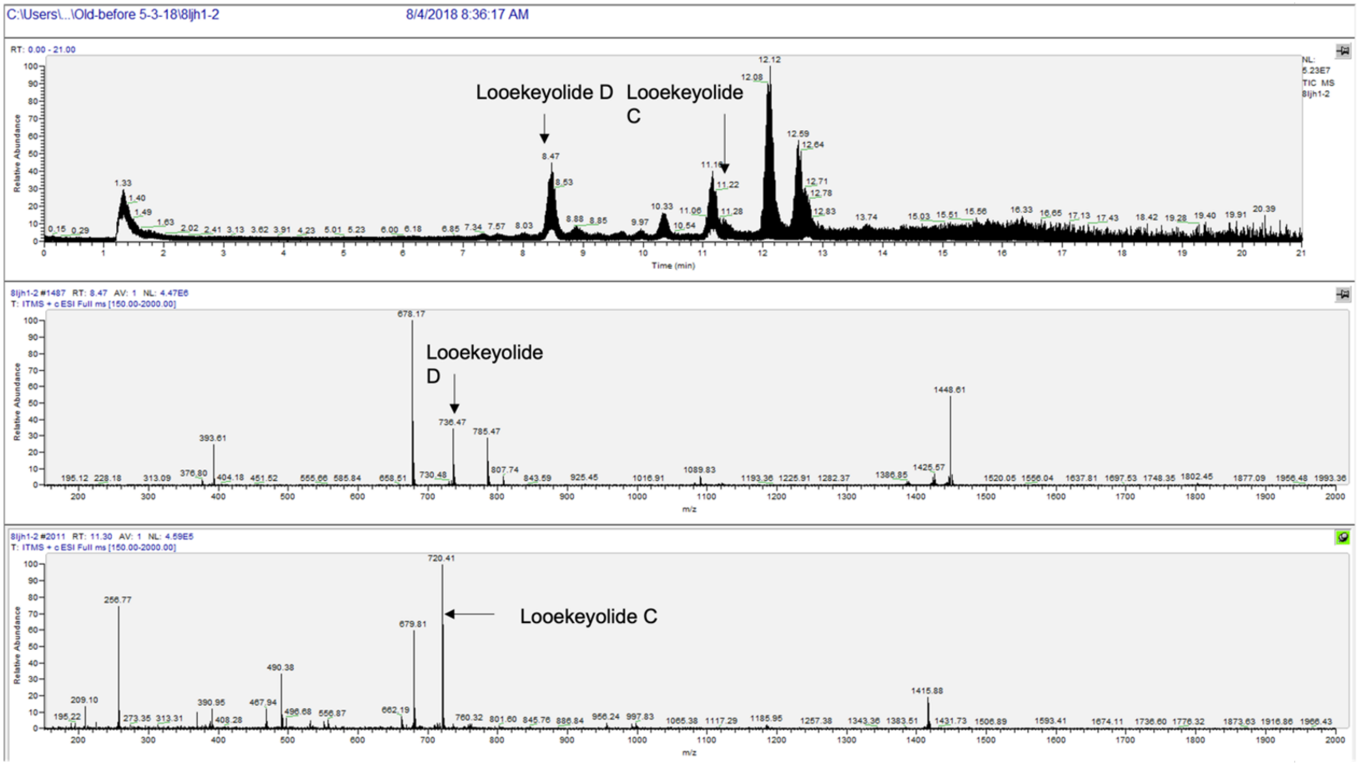

Figure S3: LRLC-MS data showing the presence of looekeyolides C and D in the EtOAc-MeOH (1:1) extract of 2018 Fort Lauderdale, Florida collection.

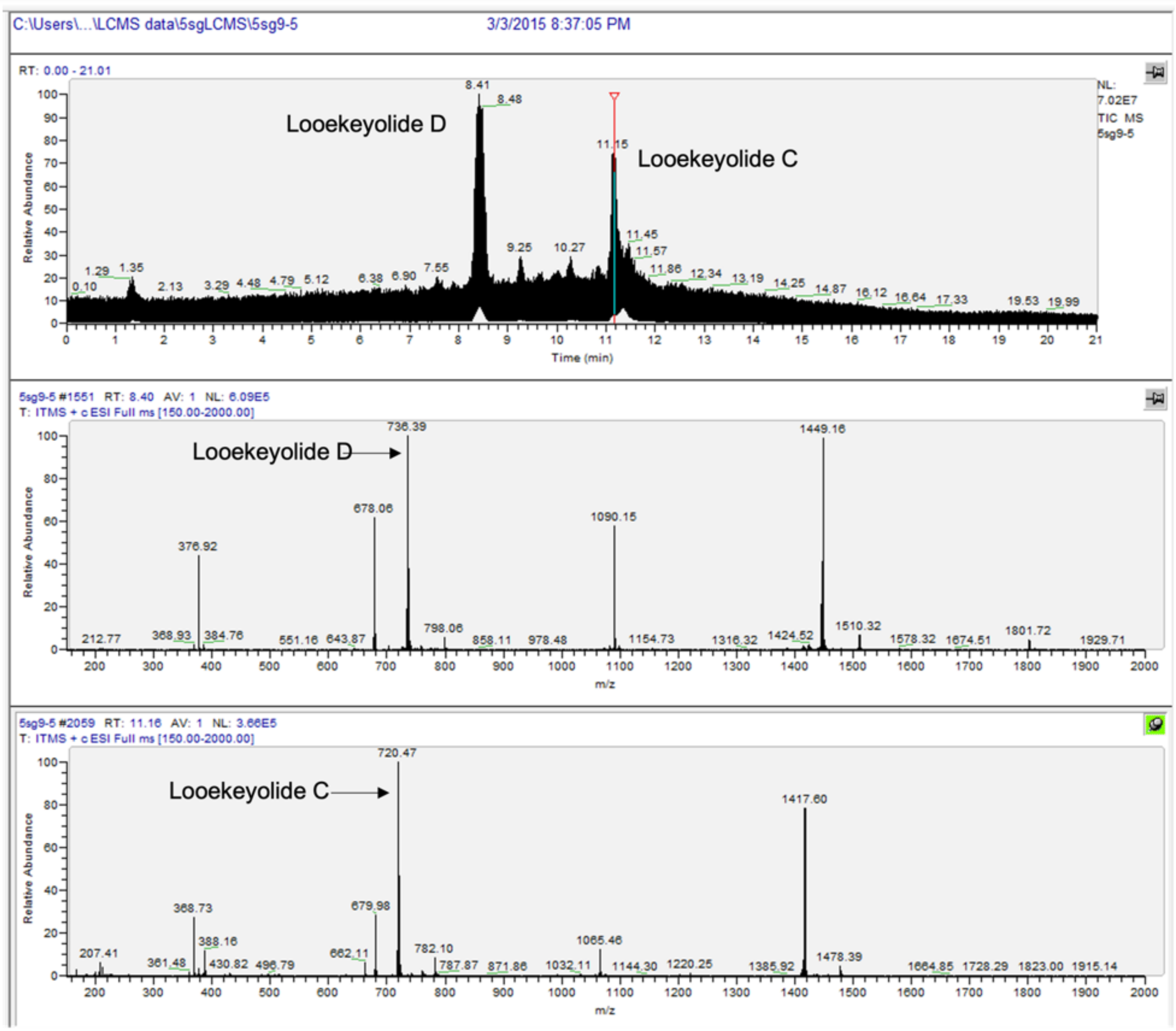

Figure S4: LRLC-MS data showing the presence of looekeyolides C and D in fraction 3 of the 2014 Belize collection.

Contents: Page #

Figure S5: ^{1}H NMR (600 MHz, CD_3OD) spectrum of looekeyolide D (**4**) S9

Figure S6: ^{13}C NMR (151 MHz, CD_3OD) spectrum of looekeyolide D (**4**) S10

Figure S7: DQF-COSY NMR (600 MHz, CD_3OD) spectrum of looekeyolide D (**4**) S11

Figure S8: HSQC NMR (600 MHz, CD_3OD) spectrum of looekeyolide D (**4**) S12

Figure S9: HMBC NMR (600 MHz, CD_3OD) spectrum of looekeyolide D (**4**) S13

Figure S10: 2D-NOESY NMR (600 MHz, CD_3OD) spectrum of looekeyolide D (**4**) S14

Figure S5: ^{1}H NMR (600 MHz, CD_3OD) spectrum of looekeyolide D (**4**)

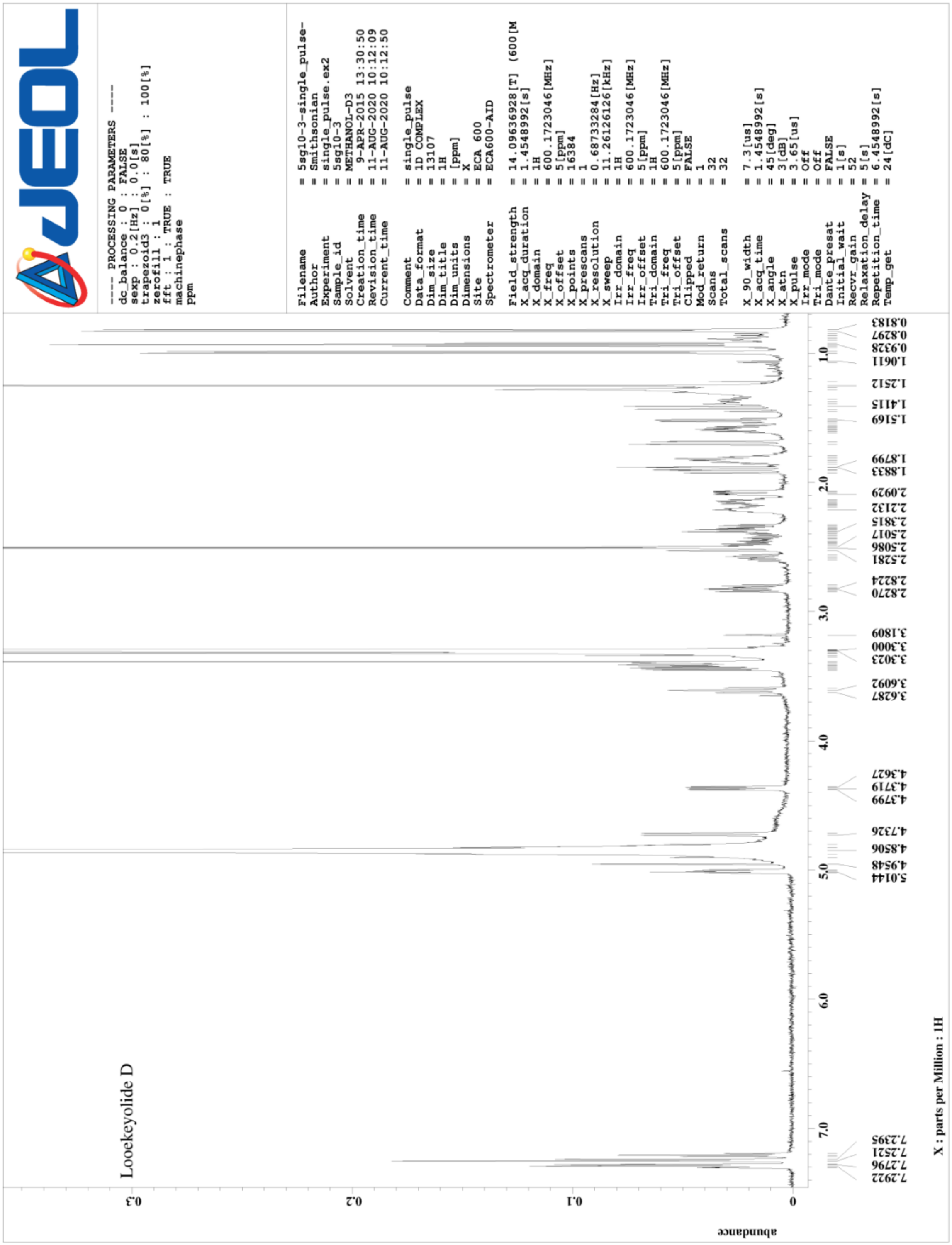

Meyer *et al.*, Cryptic diversity of Black Band Disease cyanobacteria in *Siderastrea siderea* corals revealed by chemical ecology and comparative genome-resolved metagenomics

Figure S6: ^{13}C NMR (151 MHz, CD_3OD) spectrum of looekeyolide D (**4**)

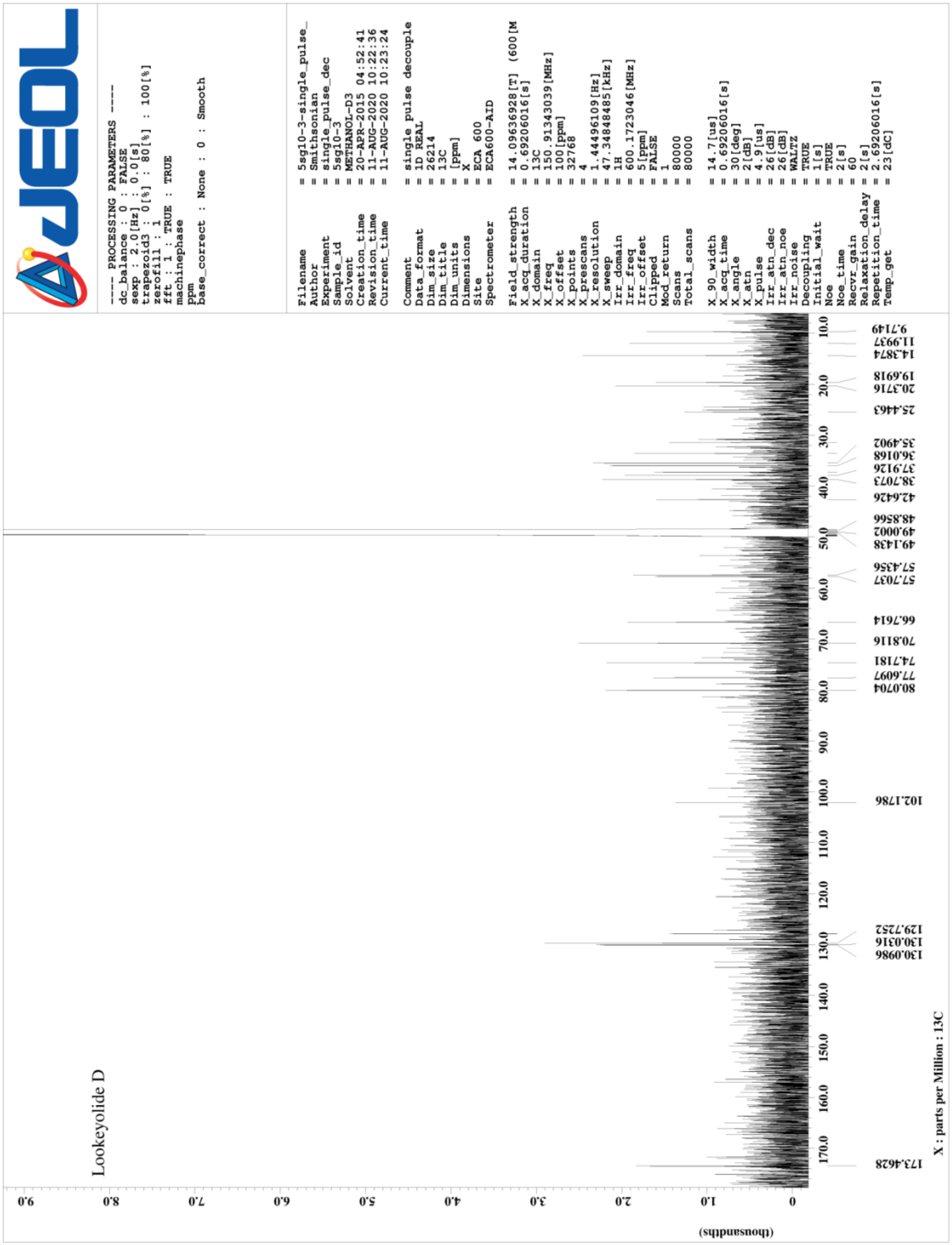

Figure S7: DQF-COSY NMR (600 MHz, CD_3OD) spectrum of looekeyolide D (**4**)

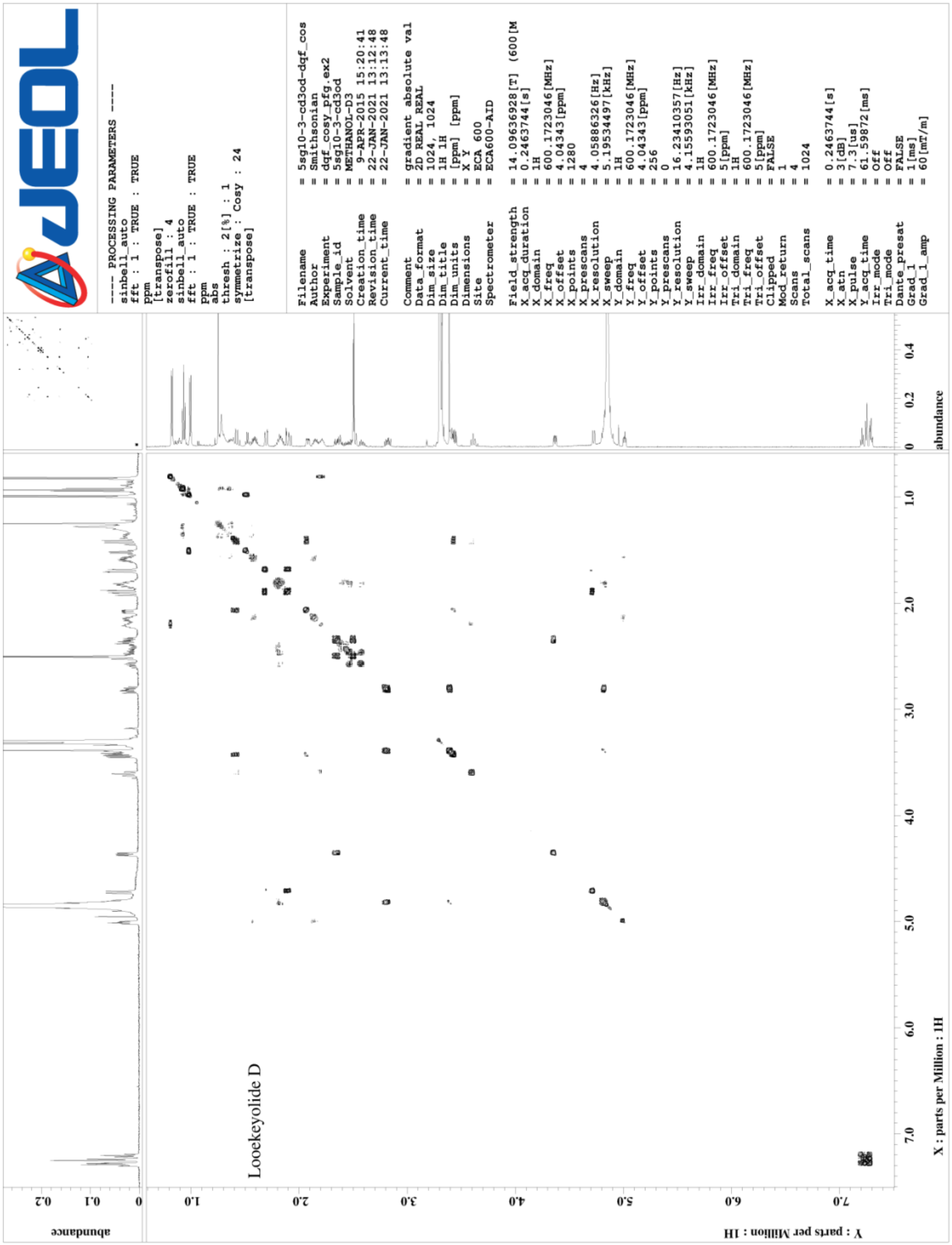

Meyer *et al*., Cryptic diversity of Black Band Disease cyanobacteria in *Siderastrea siderea* corals revealed by chemical ecology and comparative genome-resolved metagenomics

Figure S8: HSQC NMR (600 MHz, CD_3OD) spectrum of looekeyolide D (**4**)

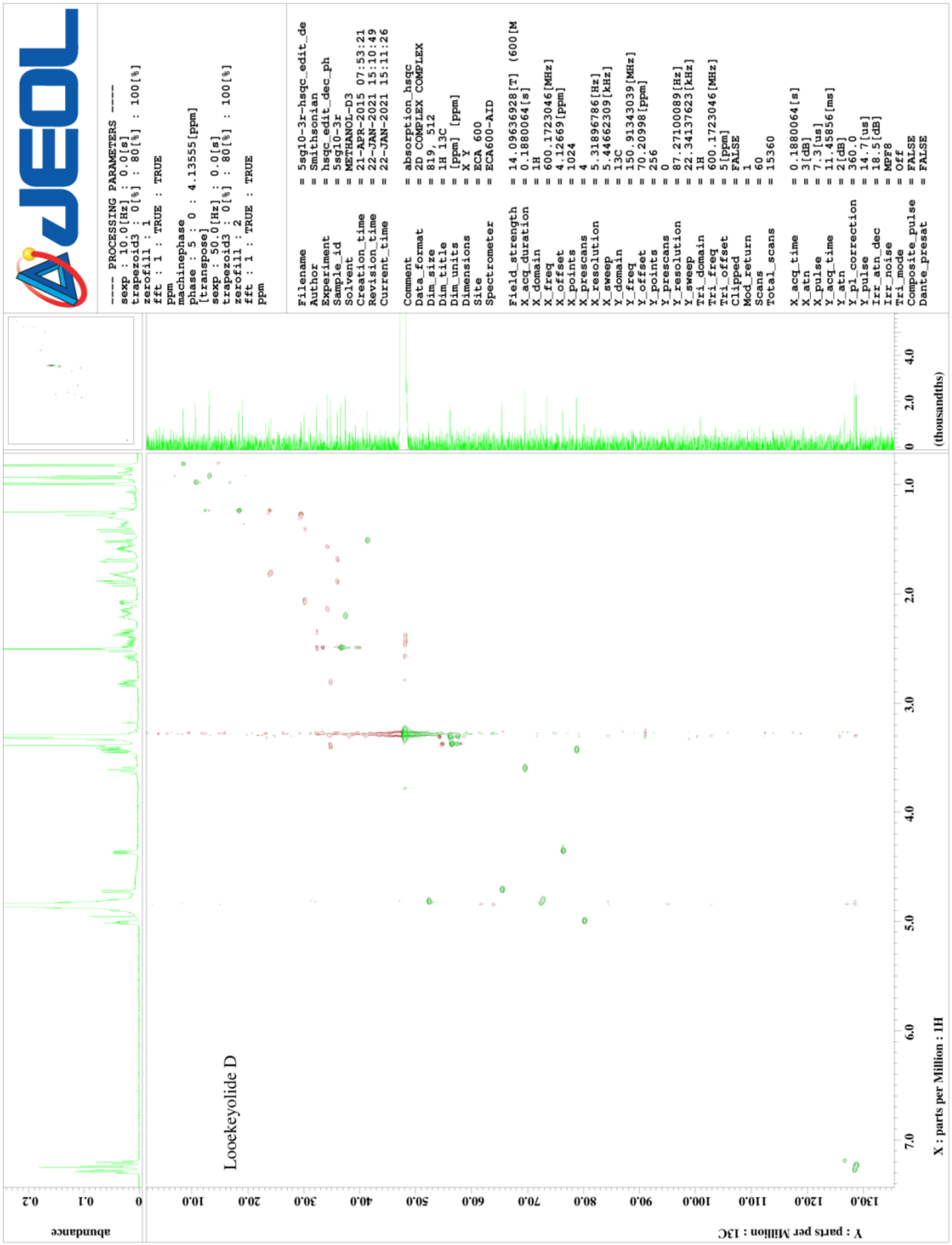

Figure S9: HMBC NMR (600 MHz, CD_3OD) spectrum of looekeyolide D (**4**)

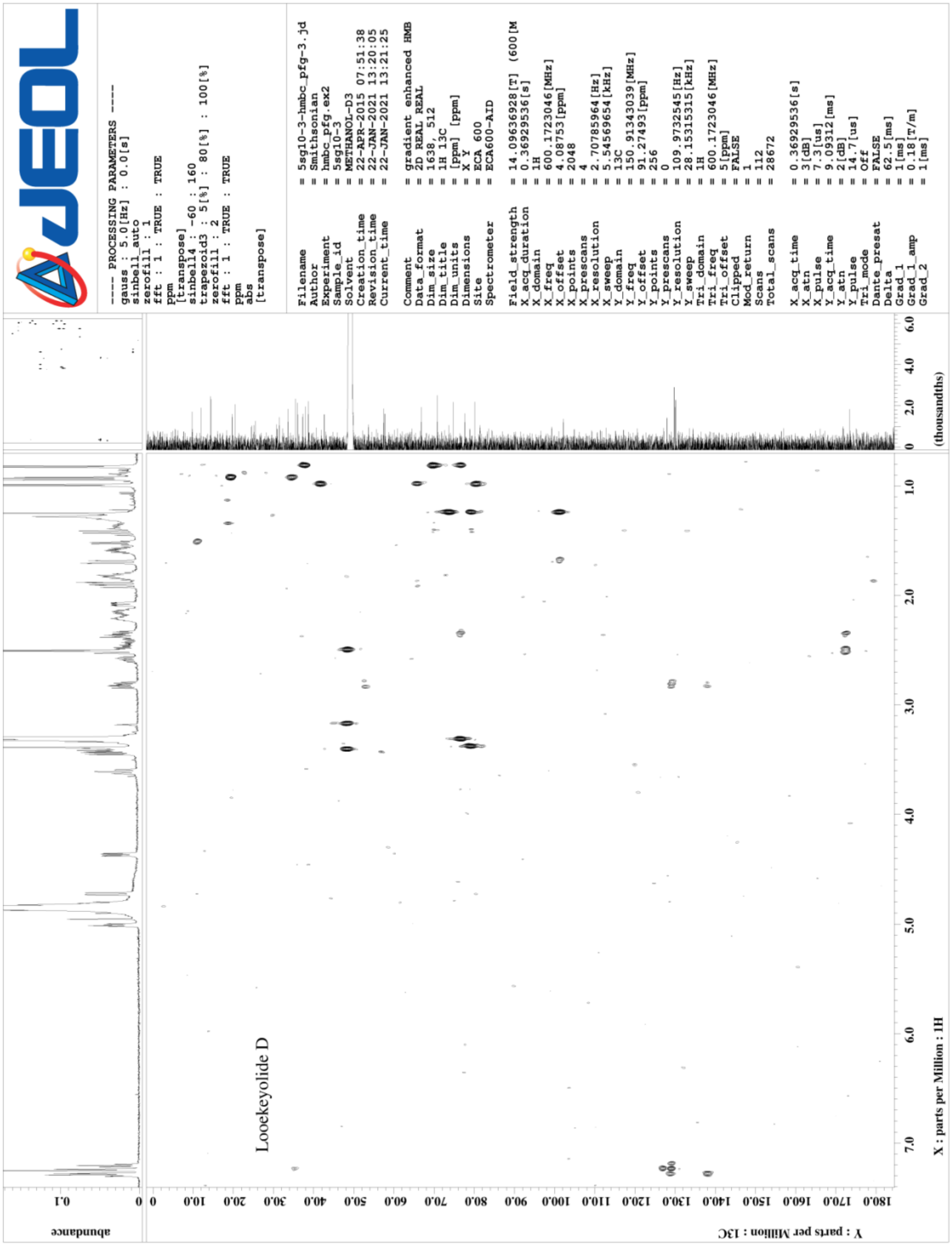

Figure S10: 2D-NOESY NMR (600 MHz, CD_3OD) spectrum of looekeyolide D (**4**)

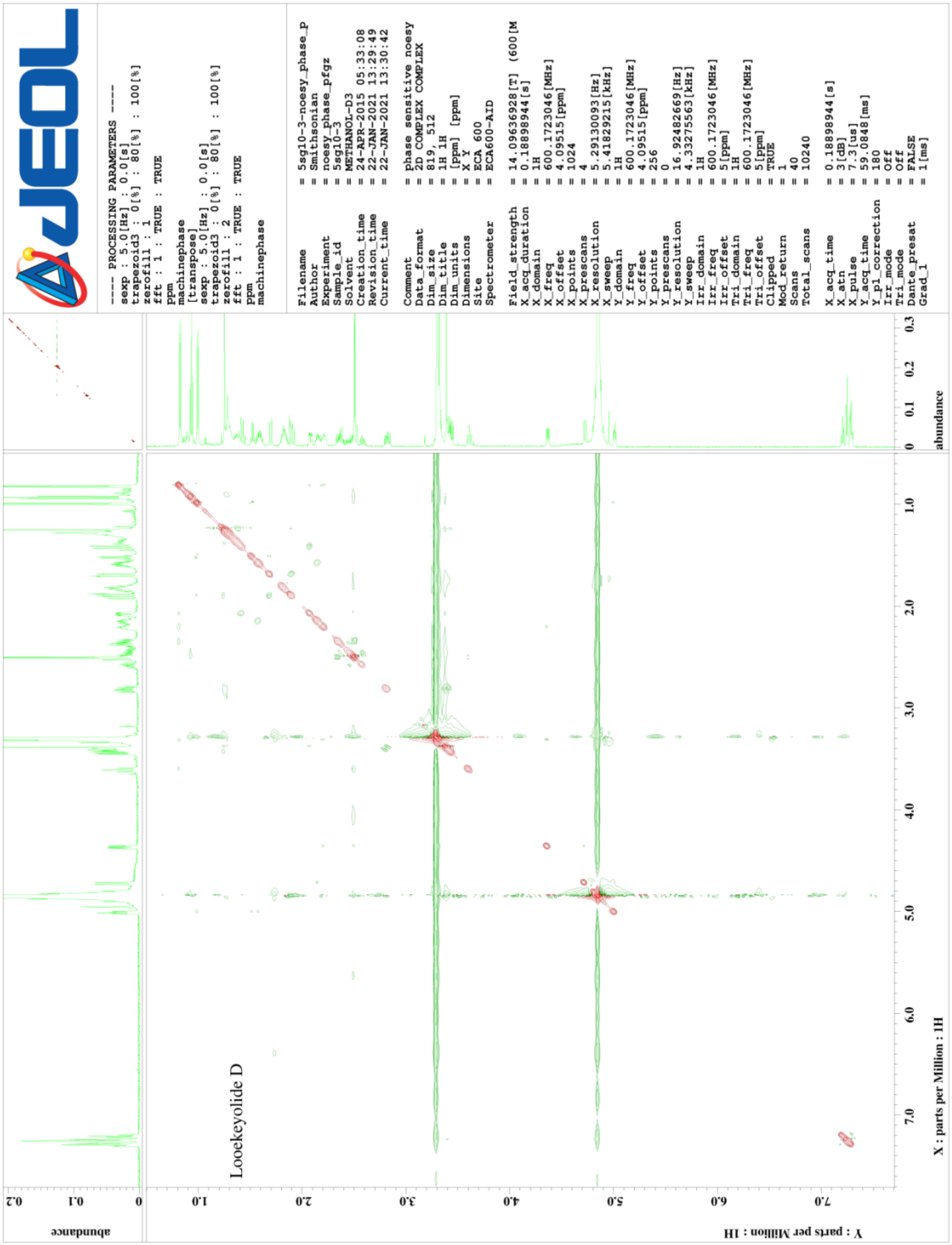

Table S1. Metadata and sequencing read metrics for V6 amplicon libraries from Black Band Disease cyanobacterial mats from *Siderastrea siderea* corals in Belize

SRA accession	Sample	Fraction	Coral	Metagenome Pool	Looekeyolide C/D	Raw reads	Quality-filtered reads
SAMN15502381	SID1	DNA	1	1	nonproducer	119,322	56,442
SAMN15502382	SID1r	RNA	1	n/a	nonproducer	299,249	295,333
SAMN15502383	SID2	DNA	2	1	nonproducer	80,827	71,330
SAMN15502384	SID2r	RNA	2	n/a	nonproducer	206,090	191,909
SAMN15502385	SID3	DNA	3	1	nonproducer	63,264	41,382
SAMN15502386	SID3r	RNA	3	n/a	nonproducer	222,750	210,397
SAMN15502387	SID8m	DNA	8m	2	nonproducer	26,671	15,743
SAMN15502388	SID8mr	RNA	8m	n/a	nonproducer	196,599	144,306
SAMN15502389	SIDa	DNA	a	2	nonproducer	49,036	31,063
SAMN15502390	SIDar	RNA	a	n/a	nonproducer	49,329	34,400
SAMN15502391	SIDE	DNA	E	2	nonproducer	35,035	16,018
SAMN15502392	SIDEr	RNA	E	n/a	nonproducer	206,638	82,614
SAMN15502393	SIDH	DNA	H	3	producer	75,558	46,112
SAMN15502394	SIDHr	RNA	H	n/a	producer	249,500	119,415

SAMN15502395	SIDI	DNA	I	3	producer	84,467	44,376
SAMN15502396	SIDIr	RNA	I	n/a	producer	218,805	71,634
SAMN15502397	SIDL	DNA	L	3	producer	9,373	2,973
SAMN15502398	SIDLr	RNA	L	n/a	producer	190,547	91,907
SAMN15502399	SIDO	DNA	O	3	producer	81,095	58,461
SAMN15502400	SIDOr	RNA	O	n/a	producer	226,302	189,228

Table S2. Metadata and sequencing read metrics for metagenomic libraries from Black Band Disease cyanobacterial mats from *Siderastrea siderea* corals in Belize and Florida

SRA Accession	Metagenome Name	Quality-filtered read pairs	Cyanobacterial MAGs	Collection site and date
SAMN15583061	SID1	33,319,838	*Roseofilum* sp. SID1.26	Carrie Bow Cay, Belize, Sept 2015
SAMN15583062	SID2	26,242,408	*Roseofilum* sp. SID2.16, *Geitlerinema* sp. SID2.20	Carrie Bow Cay, Belize, Sept 2015
SAMN15583063	SID3	23,721,563	*Roseofilum* sp. SID3.16	Carrie Bow Cay, Belize, Sept 2015
SAMN15583059	SBFL	14,889,967	*Roseofilum* sp. SBFL6	offshore reef near Ft. Lauderdale, FL, USA, July 2018
SAMN15583058	SBC	32,151,862	*Geitlerinema* sp. SBC9	enrichment culture of cyanobacterial mat from offshore reef near Ft. Lauderdale, FL, USA, July 2018
SAMN15583060	SBLK	13,355,562	Spirulinaceae bacterium SBLK1	Looe Key reef, Florida Keys National Marine Sanctuary, FL, USA, July 2017

Table A3. Quality metrics of metagenome-assembled genomes (MAGs) of A) non-*Roseofilum* cyanobacteria from *Siderastrea siderea*, B) *Roseofilum* from *Siderastrea siderea*, and C) previously published *Roseofilum* strains

A) non-*Roseofilum* cyanobacteria from *Siderastrea siderea*

GenBank Accession	MAG	Completeness	Contamination	Quality
**JAGHZO000000000**	***Geitlerinema* sp. SBC9**	**94.6%**	**4.5%**	**High**
**JAGHZP000000000**	***Geitlerinema* sp. SID2.20**	**99.1%**	**4.7%**	**High**
**JAGHZQ000000000**	**Spirulinaceae bacterium SBLK1**	**94.6%**	**5.4%**	**High**

B) *Roseofilum* cyanobacteria from *Siderastrea siderea*

GenBank Accession	MAG	Completeness	Contamination	Quality
**JAGHZH000000000**	***Roseofilum* sp. SBFL6**	**91.9%**	**4.5%**	**High**
**JAGHZI000000000**	***Roseofilum* sp. SID1.26**	**92.5%**	**4.7%**	**High**
**JAGHZJ000000000**	***Roseofilum* sp. SID2.16**	**94.3%**	**3.8%**	**High**
**JAGHZK000000000**	***Roseofilum* sp. SID3.16**	**93.4%**	**3.8%**	**High**

C) *Roseofilum* cyanobacteria from other coral species

GenBank Accession	MAG	Completeness	Contamination	Quality
**JAGHZL000000000**	***Roseofilum* sp. BLZ4**	**92.8%**	**4.5%**	**High**
**JAGHZM000000000**	***Roseofilum* sp. BLZD**	**91.0%**	**4.5%**	**High**
**JAGHZN000000000**	***Roseofilum* sp. Guam**	**93.7%**	**4.5%**	**High**
**MLAW00000000**	***Roseofilum* sp. AO1**	**94.6%**	**5.4%**	**High**
**N/A**	***Roseofilum* sp. Cya2**	**94.6%**	**5.4%**	**High**

Table S4. A) Pairwise Average Nucleotide Identity (ANI) of shared genes among cyanobacterial MAGs from Black Band Disease on *Siderastrea siderea* corals. Values of 75% or below are too close to the detection limit for confident assessments. Values above this threshold are highlighted in green.

	SBC9	SBFL6	SBLK1	SID1.26	SID2.16	SID2.20	SID3.16
SBC9							
SBFL6	73.04*						
SBLK1	72.75*	74.92*					
SID1.26	73.00*	99.87	75.00*				
SID2.16	72.79*	99.86	75.24*	99.88			
SID2.20	99.77	72.95*	72.69*	72.89*	72.99*		
SID3.16	72.67*	99.87	75.01*	99.92	99.96	72.77*	

*** too close to detection limit to be reliable**

Table S4. B) Pairwise Average Nucleotide Identity (ANI) of shared genes among *Roseofilum* MAGs from Black Band Disease on multiple coral species

	SBFL6	SID1.26	SID2.16	SID3.16	BLZ4	BLZD	Guam	AO1	Cya2
SBFL6									
SID1.26	99.87								
SID2.16	99.86	99.88							
SID3.16	99.87	99.92	99.96						
BLZ4	98.22	98.24	98.26	98.25					
BLZD	98.23	98.25	98.26	98.26	99.78				
Guam	94.64	94.63	94.61	94.65	94.68	94.62			
AO1	94.52	94.55	94.53	94.57	94.57	94.52	98.09		
Cya2	94.47	94.25	94.51	94.55	94.59	94.6	97.56	97.38	

Table S5. Specificity codes for adenylation (A) domains of two NRPSs for the biosynthesis of Lk-A/B and Lk-C/D.

A Domain	235	236	239	278	299	301	322	330	331	517	Specificity
LklG_A	G	L	F	W	I	G	A	S	G	K	2-ketoacid
LklG_A*	G	L	F	W	I	G	A	S	G	K	2-ketoacid
LklI_A	D	A	W	F	L	G	N	V	V	K	L-Leucine
LklI(F)_A	D	A	W	T	I	A	A	V	C	K	L-Phenylalanine
GrsA#	D	A	W	T	I	A	A	I	C	K	L-Phenylalanine

∗ LklG-A from the gene cluster from Lk-C; # GrsA: gramicidin S synthase 1.

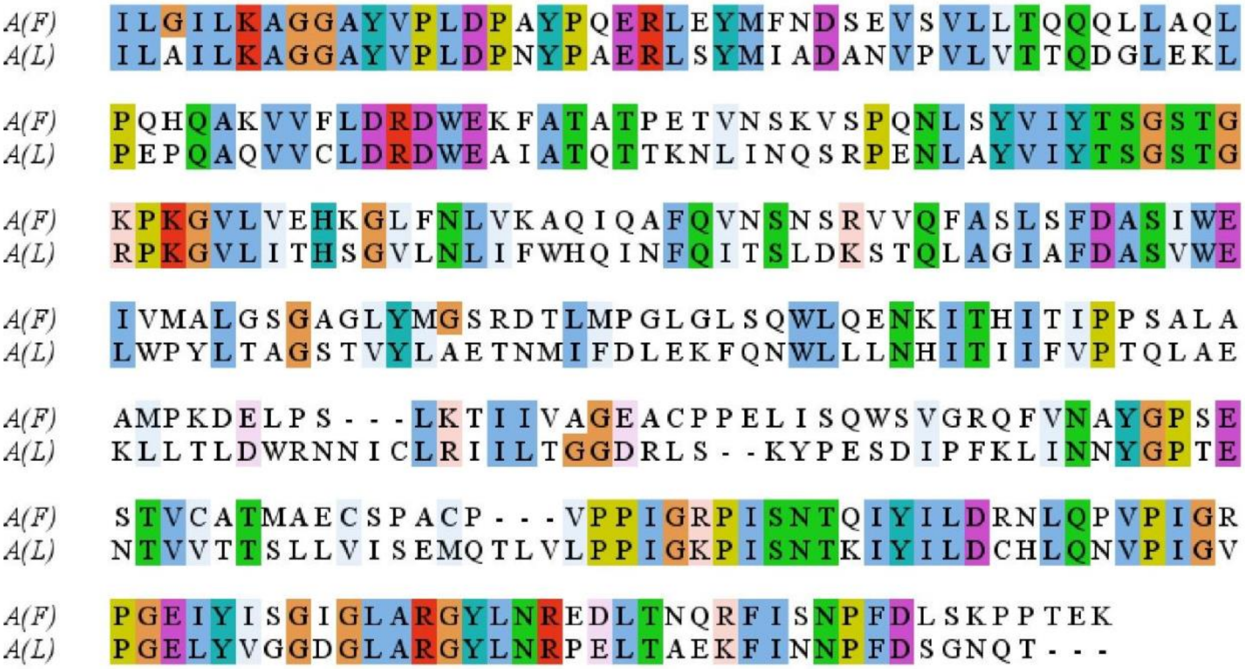

Figure S11. Alignment of A domains of LklI and LklI(F) by ClustalW[1]. These two domains showed 46% amino acid identities, significantly lower than other domains of LklI and LklI(F). The alignment figure was created using Jalview[2].

[1]Cluster W: Nucleic Acids Res. 1994 Nov 11; 22(22): 4673–4680.

[2]Jalview: Waterhouse, A.M., Procter, J.B., Martin, D.M.A, Clamp, M. and Barton, G. J. (2009) "Jalview Version 2 - a multiple sequence alignment editor and analysis workbench" Bioinformatics25 (9) 1189-1191 doi: 10.1093/bioinformatics/btp033

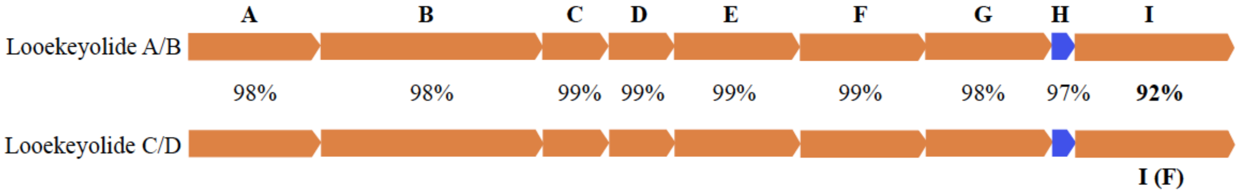

Figure S12. Comparison of gene similarities in the biosynthetic gene clusters for looekeyolide A/B and looekeyolide C/D. The two clusters for the biosynthesis of Lk-A/B (top) and Lk-C/D (bottom) show at least 97% amino acid identities, except the last NRPS. LklI shares a 92% amino acid identity with LKlI (F) and the identity deviation originates from the low identity (46%) of their A domains.

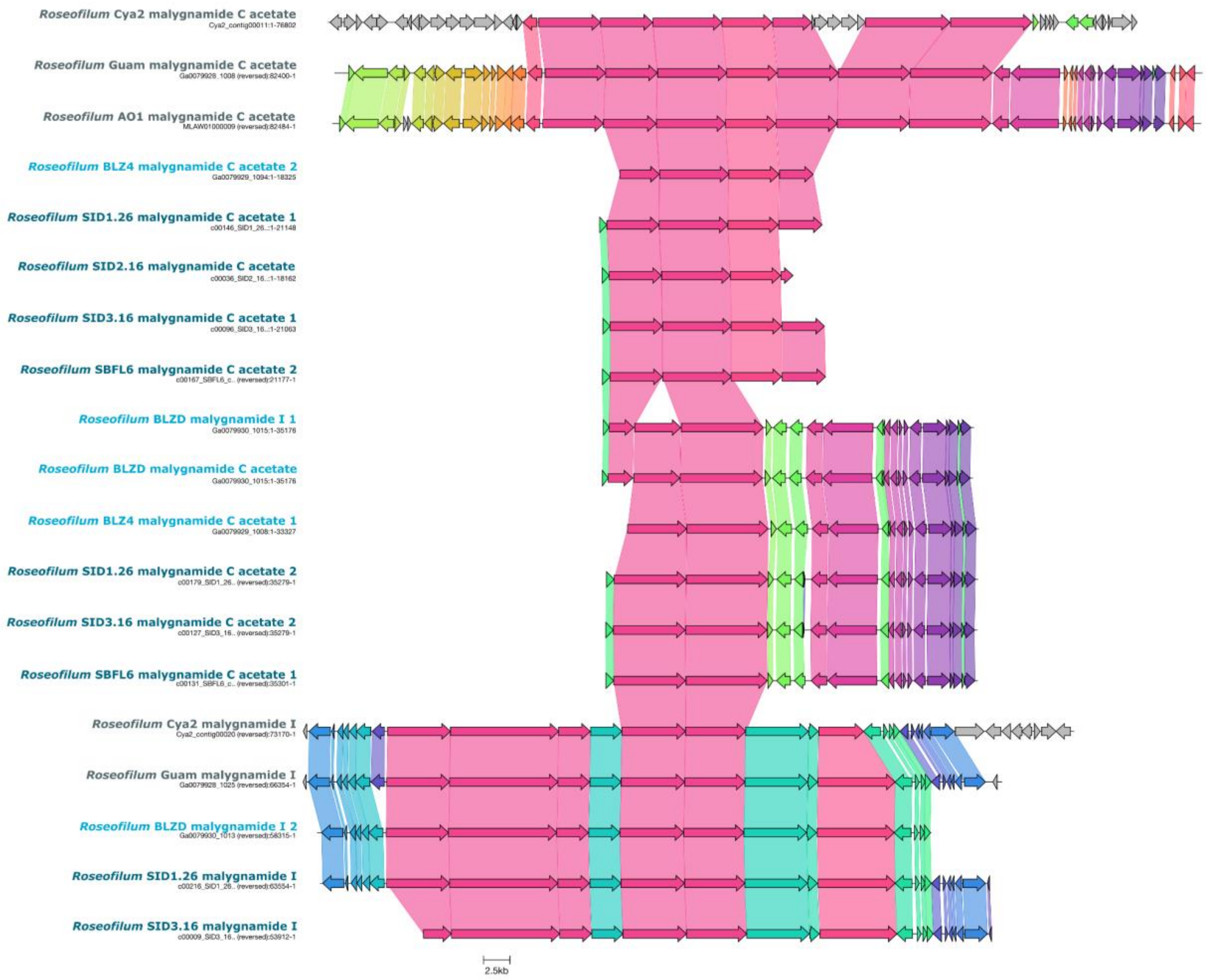

Figure S13. Biosynthetic gene clusters for malyngamides in *Roseofilum* MAGs. Biosynthetic gene clusters were annotated as malyngamide C acetate and malyngamide I by antiSMASH v 6.0. MAG names are colored by whole-genome clustering (as in Figures 2 and 3) where Caribbean *Roseofilum* from *Siderastrea* corals are dark blue, Caribbean *Roseofilum* from other coral species are light blue, and Pacific *Roseofilum* are dark grey.
